# Machine learning prediction of compressive strength in 3d printed fiber reinforced concrete using support vector regression and artificial neural networks with shapley additive explanations

**DOI:** 10.1038/s41598-026-58697-3

**Published:** 2026-06-25

**Authors:** Mohamed Abdellatief, Ahmed M. Saqr

**Affiliations:** 1 Department of Civil Engineering, Higher Future Institute of Engineering and Technology in Mansoura, Mansoura, Egypt; 2 Irrigation and Hydraulics Department, Faculty of Engineering, Mansoura University, Mansoura, 35516 Egypt

**Keywords:** 3D-printing, Machine learning, Support vector regression, Compressive strength, Shapley additive explanations, Fiber-reinforced concrete, Engineering, Materials science, Mathematics and computing

## Abstract

The swift progression of three-dimensional (3D) concrete printing has opened the door to the creation of innovative materials, such as fiber-reinforced concrete, making it essential to develop accurate models for predicting their mechanical properties. Accurately estimating the compressive strength (CS) of such materials is required for optimizing mix designs and ensuring structural performance. In this context, the present study is the first to systematically investigate and compare three different support vector regression (SVR) kernels, namely, linear SVR (L-SVR), polynomial SVR (Poly-SVR), and radial basis function SVR (RBF-SVR), for predicting the compressive strength of normal, high, and ultra-high strength 3D-printed fiber-reinforced concrete (3DPFRC). A model was developed and validated using a dataset of 278 samples collected from the literature. In addition to SVR models, Artificial Neural Networks (ANN) and Gradient Boosting Machines (GBM) were developed for benchmarking purposes. Results from statistical evaluation revealed that the ANN model achieved the highest predictive accuracy overall, outperforming the L-SVR, Poly-SVR, and GBM models. Among the SVR-based models, the RBF-SVR demonstrated superior performance, showing competitive accuracy with the ANN while outperforming both the other SVR variants (L-SVR and Poly-SVR) as well as the GBM model in terms of coefficient of determination (R²) value and error metrics. All models achieved R² scores ranging between 0.94 and 0.86 for the training datasets and 0.93 and 0.72 in the testing datasets. The RBF-SVR model, in particular, offered an excellent balance between accuracy, training speed, and reliability, making it a strong and efficient alternative to more complex models. Additionally, SHapley Additive exPlanations (SHAP) analysis identified the water-to-cement ratio, silica fume content, and cement volume as the most influential parameters affecting compressive strength.

**Supplementary Information:**

The online version contains supplementary material available at 10.1038/s41598-026-58697-3.

## Introduction

The traditional construction industry confronts major problems because of its lack of workers and the high amount of formwork waste it produces, as well as the growing impact on the environment^1,2^. The sector needs to implement advanced building techniques if it wants to establish environmental sustainability measures. The construction industry has achieved a groundbreaking milestone through 3D concrete printing, which modifies conventional construction practices through additive manufacturing. The approach delivers various benefits, which include automatic construction procedures and formwork elimination alongside reduced manual labor requirements and enhanced productivity and environmental^3–5^. The automated concrete nozzle system allows 3D-printed concrete (3DPC) to deposit material in layers according to digital model instructions without extensive human intervention. The ability to print directly without formwork enables 3DPC to create complex geometric structures that would otherwise be impossible to achieve through standard building approaches. 3DPC shows substantial promise, but its advancement remains restricted by material weaknesses that emerge from the natural brittleness of standard concrete blends^6–9^. Furthermore, the sustainability of 3D concrete printing is increasingly linked to the upcycling of plastic waste, as highlighted by Singh et al.^10^, who emphasized the potential of recycled polyolefins in natural fiber and sustainable filler-based biocomposites for additive manufacturing applications. The use of steel reinforcement is essential to avoid brittle or quasi-brittle failures in building structures. However, the implementation of steel reinforcement during 3DPC construction faces major obstacles because of the restrictions that come from the layer-based printing method. The current reinforcement techniques create additional difficulties in the construction workflow and reduce the automation benefits of 3DPC^4,5,11^. Various short-cut fibers have been studied by researchers to combine with concrete to produce fiber-reinforced concrete (FRC) that has better printability characteristics. The reinforcement of concrete structures commonly uses polyvinyl alcohol (PVA), polyethylene (PE), carbon, glass, basalt, steel, and polypropylene (PP) fibers. The enhanced FRC materials through these advancements help 3DPC systems deliver superior mechanical properties without sacrificing their automated operation and design flexibility^2,8,9,12^.

3D-printed fiber-reinforced concrete (3DPFRC) quality assessment relies heavily on its compressive strength (CS) measurement. Research reveals that 3DPFRC materials, including high-performance concrete (HPC) and conventional 3DPC, have exhibited substantial CS enhancement compared to conventional concrete (CC)^2,5,9^. Research demonstrates that 3DPFRC types with steel fibers in ultra-high-performance concrete (UHPC) achieve the maximum CS levels of 156 MPa^11,13–15^. The creation of HPC and UHPC, and CC through locally sourced supplementary cementitious materials, including fly ash (FA), metakaolin, silica fume (SF), enabled the development of materials with better mechanical performance. For the three concrete types, the desired 28-day CS values were 120 MPa for UHPC^13–15^, 50 MPa for HPC, and 35 MPa for CC^10,11,16,17^. The mixture of these materials successfully passed the extrusion-based 3D printer test to assess their printability. The 3DPFRC receives evaluation through CS as a fundamental parameter. When examining the CS of 3DPFRC alongside other 3DPFRC, such as HPC and traditional 3DPC, a comparison demonstrates substantial improvements in CS compared to CC^2,5,11^. The maximum CS value in 3DPFRC belongs to UHPC with steel fibers, which reaches 156 MPa^11,13–15^. An advanced method has been implemented to manufacture three different 3DPFRC types, including HPC, UHPC, and CC that use locally available supplementary cementitious materials such as slag and FA, and metakaolin and SF to achieve advanced mechanical properties. The performance of UHPC and HPC improved when they contained 6 mm steel fibers at 2% and 1.5% levels, while 8 mm PVA fibers at 0.75% dosage optimized CC characteristics^11,13,16,17^. The CS of 3DPFRC depends on multiple variables, such as internal mix design elements and raw materials, as well as fiber length and volume, and geometric shapes, while external factors include curing time and loading direction^13,16,18–21^. The current knowledge base about how these elements affect the mechanical performance of 3DPFRC is restricted. The prediction of UHPC-based 3DPFRC CS lacks any existing analytical models or empirical equations that require expensive and time-consuming experimental verification^13,15,22–24^.

Machine learning (ML) techniques based on data analysis show great potential for forecasting the CS of 3DPFRC while analyzing complex relationships between different input parameters^22^. Multiple studies have confirmed ML models as effective tools to predict CS values for various FRC types^25,26^. Different FRC types show accurate CS prediction results through the use of SVR models and RF models, as well as GBM models^22,25,26^. Through their implementation in ML models, scientists can improve the precision of their CS predictions and uncover complex connections between mix proportion, fiber type, and curing conditions, which remain difficult to identify using standard testing practices. The various engineering applications demonstrate the successful implementation of ML models. Shehadeh et al.^27^ found that GBM provides faster predictions than XGBoost for estimating residual values of heavy construction equipment. Lee et al.^28^ also found that CatBoost significantly surpassed XGBoost, SVR, and GBM models in predicting the strength of tubular steel columns loaded with concrete. Additionally, the Shapley Additive Explanations (SHAP) framework uses game theory principles to evaluate model feature dependency^26^. ML and optimization methods have proven their efficiency for improving 3DPFRC design and performance predictions according to recent research findings. The study by Uddin et al.^23^ evaluated six ML models and demonstrated high accuracy in predicting CS and flexural strength. Among the tested models, XGBoost, LightGBM, CatBoost, and natural gradient boosting outperformed the conventional random forest (RF) and support vector regression (SVR) models. SHAP analysis indicated that the water-to-cement ratio (W/C ratio) and fiber volume fraction were the most influential factors affecting mechanical performance. In another study, Alyami et al.^24^ enhanced the predictive accuracy of the RF model by integrating metaheuristic optimization based on the tunicate swarm algorithm. However, most existing studies mainly focus on individual ML models or single optimization techniques, highlighting the need for future research on hybrid approaches and the use of larger and more diverse datasets.

As discussed above, previous research findings clearly highlight the precision and reliability of ML algorithms in predicting 3DPFRC strength. Thus, the research goal is to address the gap in accurately predicting the CS of normal, high, and ultra-high-performance 3DPFRC by developing a robust ML model. Specifically, it explores the application of linear SVR (L-SVR), radial basis function (RBF) SVR (RBF-SVR), and polynomial SVR (Poly-SVR) for this purpose for the first time, providing a novel alternative to traditional empirical equations and existing predictive models. Although boosting algorithms such as XGBoost, LightGBM, and CatBoost have shown excellent performance in other materials, this study deliberately focuses on SVR kernels to examine whether simpler, kernel-based methods can achieve comparable predictive accuracy with reduced complexity, faster training, and improved interpretability in the context of 3DPFRC. By comparing SVR’s performance with GBM and artificial neural network (ANN) models, the study seeks to establish a reliable, efficient, and new method for improving the structural performance of 3DPFRC in modern building practices through the optimization of mix designs.

## Materials and methods

### Data collection

Recent studies indicate that the properties of 3D-printed fiber-reinforced concrete (3DPFRC) are affected by various factors, including the mix design, fiber content, printing parameters, and curing conditions, which significantly impact its mechanical performance and structural integrity. The mix designs typically include cement, sand, water-to-cement (W/C) ratio, and supplementary cementitious materials such as fly ash (FA), ground granulated blast-furnace slag (GGBS), and silica fume (SF). Fiber content, with variable length and diameter (primarily steel and polyvinyl alcohol (PVA) fibers), enhances both tensile and compressive properties. Printing parameters (e.g., nozzle diameter and layer height) and curing conditions (1–28 days) also play a critical role, resulting in a wide range of compressive strength (CS) values (8–153.4 MPa)^13,15,22–24^.

A comprehensive dataset of 278 experimental data points was systematically compiled from more than 25 peer-reviewed studies published between 2018 and 2024, as shown in Supplementary Table S1. Inclusion criteria required studies to report complete mix proportions, fiber characteristics (type, length, and diameter), curing age, and measured compressive strength values for extrusion-based 3D-printed fiber-reinforced concrete (covering normal, high-performance, and ultra-high-performance grades). Studies with incomplete data, non-extrusion printing methods, or lacking essential mix design information were excluded. Data extraction was performed manually with cross-verification to ensure accuracy. All input features were normalized using StandardScaler prior to model training to handle heterogeneity in scales and units.

**Fig. 1:**
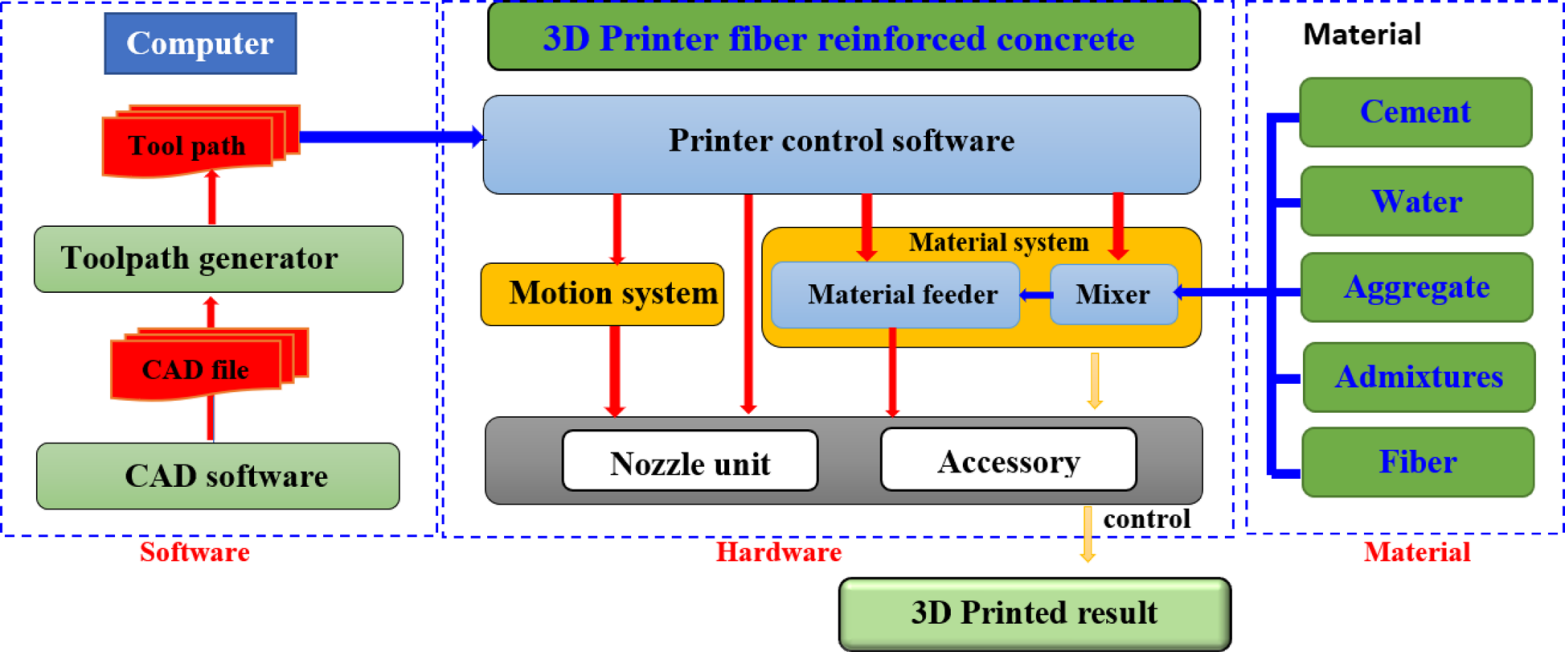
Schematic representation of the three-dimensional (3D) printing process for fiber-reinforced concrete.

As shown in Fig. 1, the typical 3D printing process is composed of three essential elements: materials, hardware, and software. The conceptual model is ultimately created through the integration of digital, mechanical, and material workflows. Factors such as nozzle diameter, layer height, printing speed, and material composition can alter the final characteristics of the printed structure^2,3,6,13,15,22,23^. In this process, the nozzle unit is responsible for material extrusion, directly shaping the printed layers. The accessory unit (Fig. 1) represents auxiliary systems such as vibration devices, heating/cooling elements, or smoothing attachments, which operate under control signals to assist in improving interlayer bonding, surface finish, or dimensional accuracy. Together, the nozzle unit and accessory system ensure that the raw material delivered by the mixer is transformed into the final 3D-printed result. For instance, higher printing speeds may lead to increased porosity and reduced mechanical strength due to less optimal bonding between layers, while adjusting the fiber content and mix proportions can enhance the material’s mechanical performance. Additionally, the printing direction and curing conditions support the assessment of the integrated density and structural integrity of the printed concrete. Thus, optimizing these production parameters is crucial for achieving the desired properties in 3DPFRC.

**Fig. 2:**
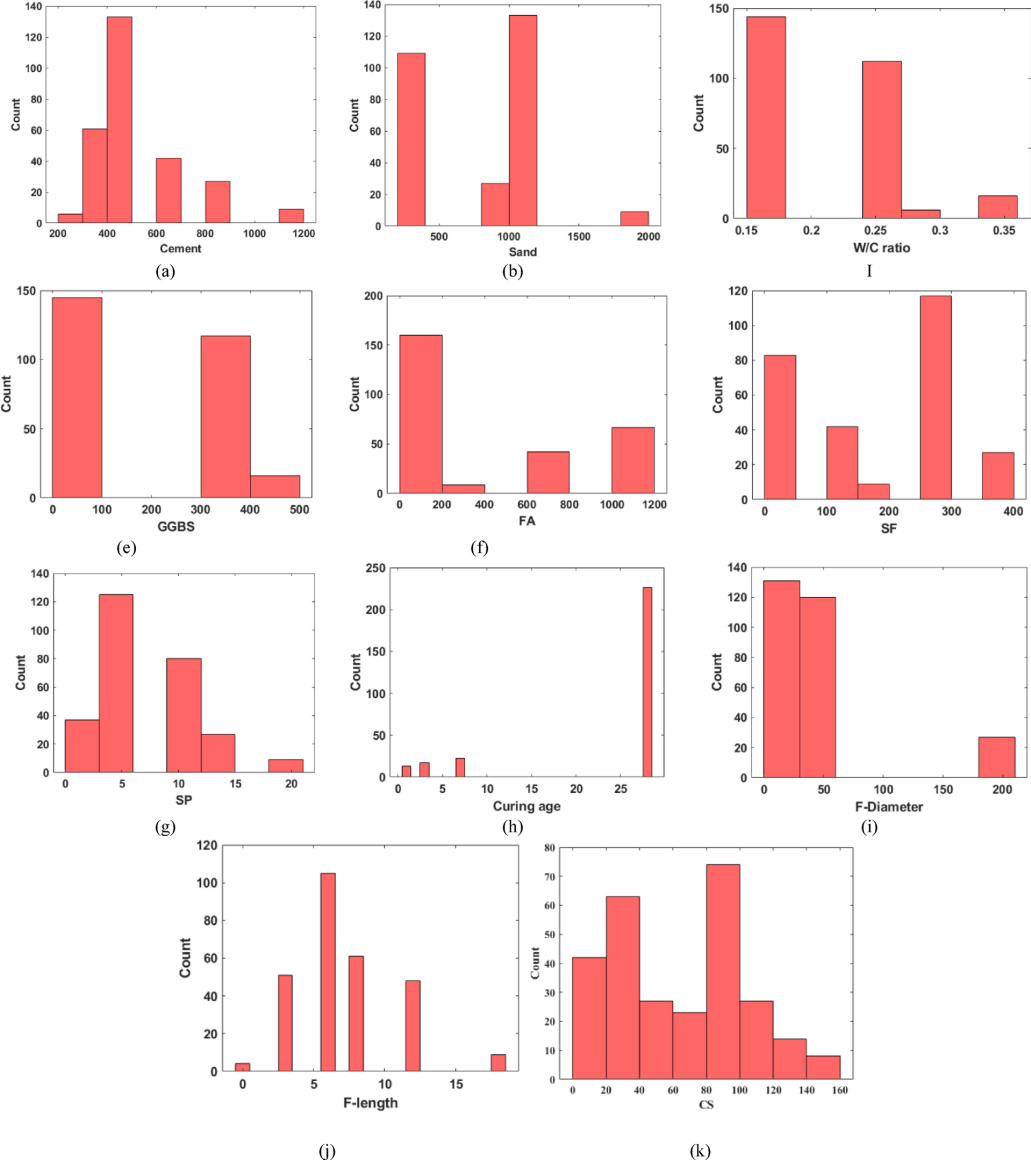
Frequency histogram of: (**a**) cement, (**b**) Sand, (**c**) water-to-cement ratio (W/C ratio), (**d**) IS, (**e**); (**f**) silica fume (SF), (**g**) superplasticizer (SP), (**h**) curing age; (**i**) Fiber diameter (F-length); (**j**) Fiber length (F-length), and (**k**) compressive strength (CS).

The dataset includes 10 input parameters: cement content, water-to-cement (W/C) ratio, fine aggregate (sand), ground granulated blast-furnace slag (GGBS), silica fume (SF), superplasticizer (SP), curing age, fiber length, and fiber diameter. To ensure the generalizability of the models, a diverse set of scenarios was collected, accounting for various material and printing conditions. The statistical distribution and characteristics of these parameters are presented in Fig. 2; Table 1^1,3–5,11,13–15,17,18,23,24,29–35^. The statistical details shown in Table 1 serve as a credible baseline for the research on machine learning (ML) model prediction of the compressive strength (CS) of 3DPFRC. All input features were normalized using StandardScaler prior to model training to handle the heterogeneity in scales and units among the variables and to improve model stability. It is worth noting that several key printing process parameters (e.g., printing speed, nozzle travel rate, extrusion pressure, and layer interval time) were not consistently reported in the collected studies and were therefore not included as input features.

**Table 1:** Characteristics of the variables (dependent and independent).

Variables	Unit	Mean	Max.	Min.	Stan dev.	Kurtosis	Skewness	Contribution
Cement	kg/m^3^	523.5	1112	285.3	196.5	1.11	1.18	Input
Sand	kg/m^3^	780.6	1902	246	416.3	–0.45	0.20	
Fly ash (FA)	kg/m^3^	351.4	1141.1	0	440.5	–0.20	0.80	
GGBS	kg/m^3^	161.4	450	0	170.9	–1.32	0.65	
Silica fume (SF)	kg/m^3^	172.3	377.8	0	131.4	–1.80	0.19	
Superplasticizer (SP)	kg/m^3^	6.47	20	0	4.9	–1.42	–0.15	
W/C ratio	–	0.21	0.35	0.15	0.05	–0.38	0.61	
Curing age	Days	23.55	28	1	9.3	0.87	–1.67	
Fiber diameter	µm mm	46.05	200	15	51.1	5.08	2.60	
Fiber length		7.23	18	0	3.52	1.09	0.90	
Compressive strength	MPa	64.89	153.4	8	38.3	–1.09	0.23	Output

**Fig. 3:**
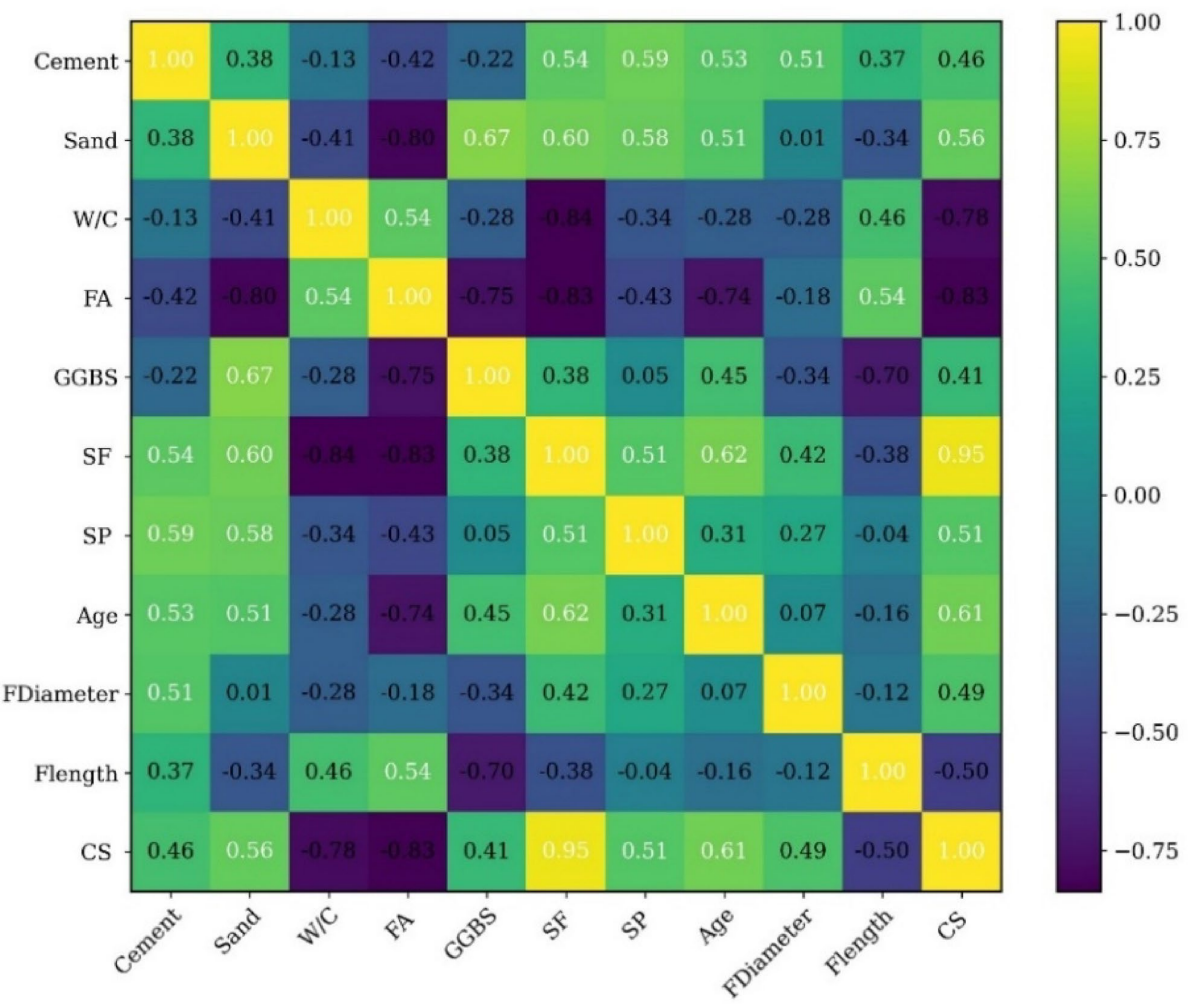
Correlation matrix between input variables and output variable.

In this study, 75% of the dataset is utilized for training, while the remaining 25% serves as the test set. This test set, consisting of unseen data, is used to find the accuracy and predictive performance of the trained ML model. The goal of the regression task is to uncover a hidden relationship between the input and output variables. Traditional regression analysis attempts to create an explicit equation involving multiple variables^19–21,36,37^. As shown in Fig. 3, cement has the highest linear Pearson correlation coefficient with CS, but the correlation is weak. This suggests that the relationship between CS and the input parameters is not a simple linear one, but rather a complex nonlinear mapping. This complexity makes it challenging to establish an explicit equation, which is why ML models are utilized in this study to predict the CS of 3DPFRC.

### Constructing predictive models

**Fig. 4:**
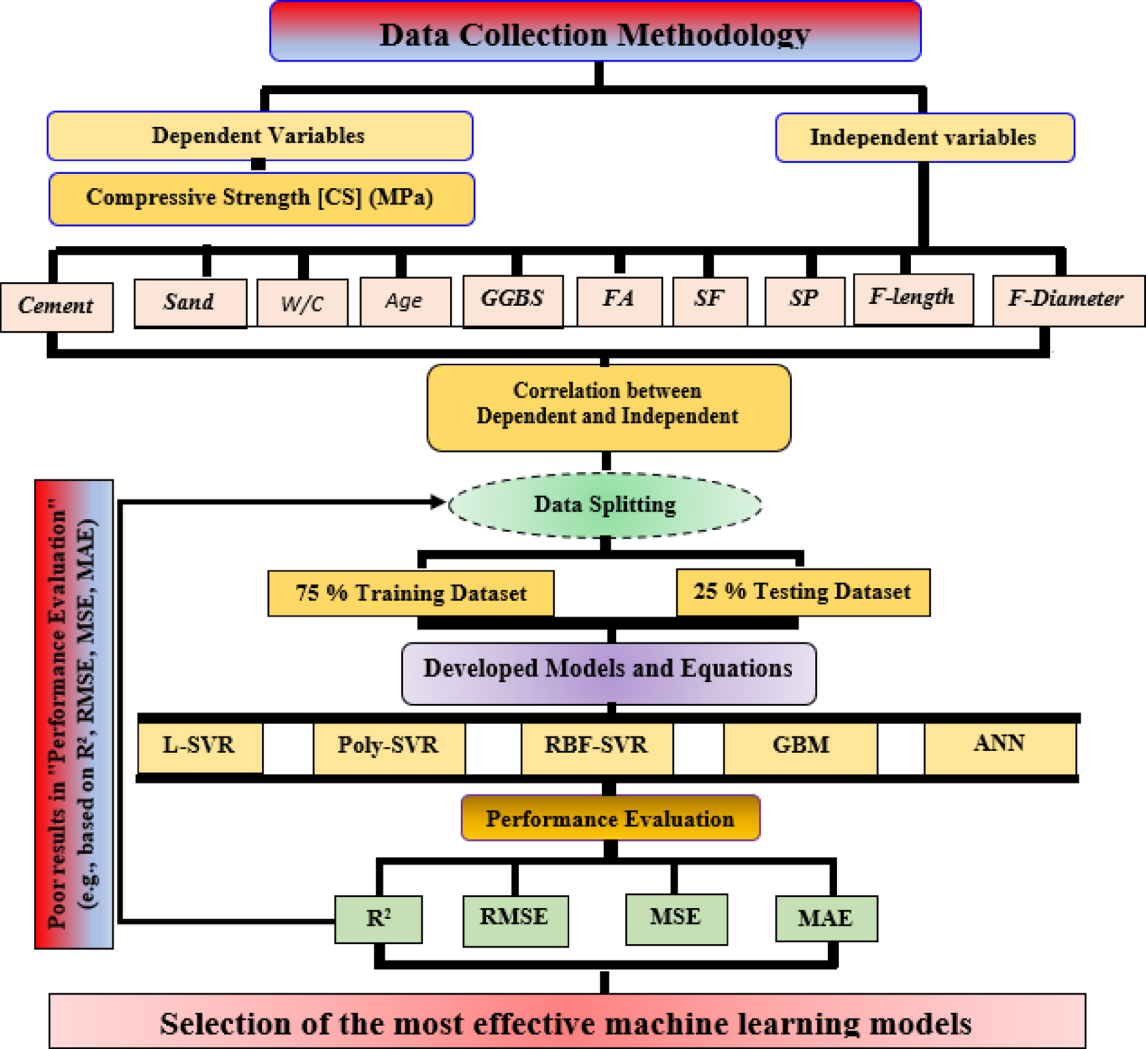
Flowchart diagram of the study procedure.

Fig. 4 shows the flowchart of the study methodology for predicting CS of 3DPFRC using the proposed ML models, which are described in the following sub-sections.

#### Support vector regression (SVR) model

SVR is an ML method designed to model different types of data relationships by using various kernel functions. The most common types of Radial Basis Function (RBF-SVR), Polynomial SVR (Poly-SVR), and SVR are Linear SVR (L-SVR). Each variant is suited to specific kinds of data patterns, with their kernel functions helping to transform input data into higher-dimensional spaces for better analysis and prediction^25,26,38^.

- **Linear SVR**.

L-SVR is particularly suited for scenarios where the correlation between predictor variables and the target outcome is presumed to be linear. Here, the kernel function used is straightforward and represented as a simple linear function (Eq. 1).

1 $$K\left( {x{\text{ }},{\text{ }}x^\prime } \right) = x \cdot x^\prime$$

where *x* and $x^{\prime}$ are the input feature vectors, and ⋅ represents the dot product. The linear model fits the data by minimizing the error margin while keeping the decision boundary as wide as possible. This is useful for predicting outcomes where the relationship between inputs and outputs is approximately linear, such as predicting material strength under controlled conditions with little to no non-linearity in the behavior. In linear SVR, the primary objective is to identify a function that predicts the target values as accurately as possible using Eq. 2.

2 $$f{\text{ }}\left( x \right){\text{ }} = {\text{ }}\left( {w{\text{ }} \times {\text{ }}x} \right){\text{ }} + {\text{ }}b$$

where *b* is the bias, *w* is the weight vector within the area of characteristic, and *x* is the input vector.

- **Polynomial SVR**.

Polynomial SVR (Poly-SVR) is used when the correlation between predictor variables and the target outcome can be approximated by a polynomial. The polynomial kernel function maps the data into a higher-dimensional space using polynomial terms of the input features. The polynomial kernel can be expressed by Eq. 3.

3 $$K{\text{ }}\left( {x{\text{ }},{\text{ }}x^\prime } \right){\text{ }} = {\text{ }}(x \cdot x^\prime {\text{ }} + {\text{ }}c)^{d}$$

where *c* is a constant, and *d* is the degree of the polynomial. Polynomial SVR functions well to handle non-linear but predictable data patterns that exist in material CS prediction problems. The polynomial function demonstrates superior data complexity understanding compared to linear kernels through its ability to capture higher-order input-output relationships^26,38–40^.

- **Radial basis function (RBF)-SVR**.

The implementation of radial basis function (RBF-SVR) occurs in cases where predictor variables show highly non-linear relationships with the target output. The RBF kernel transforms data into an infinite-dimensional feature space, which helps to understand intricate relationships. The mathematical representation of the RBF kernel function follows the pattern given in Eq. 4.

4 $$\:k\left({x}_{k}^{*}.x\right)\:=\:{exp}(-g{||{x}_{i}-{x}_{j}||}^{2})$$

The distance between input vectors is measured with the Euclidean norm $\:{||{x}_{i}-{x}_{j}||}^{2}$ while the Gaussian function’s width depends on the parameter g. The RBF SVR demonstrates strong data processing capabilities for complicated datasets that defy simple representation through linear or polynomial functions. The method becomes most beneficial when working with complex data patterns and dependencies, including concrete mixtures CS prediction, which has multiple nonlinear factor interactions^26,38–40^.

#### Gradient boosting machine (GBM)

Boosting functions as a sequential ensemble learning approach that improves model accuracy through the combination of multiple weak learners into a strong predictive model. The initial training process for the base learner begins with the original dataset. Subsequently, the distribution of the training samples is modified based on the base learner’s performance, assigning increased weights to instances that were incorrectly classified, as discussed in references^25,41–44^. This iterative procedure proceeds through multiple cycles, wherein each successive learner concentrates on rectifying the misclassifications of its predecessors. Finally, the final prediction model is created by combining all of the weak learners. The GBM model is among the most prevalent EL approaches in ML, applied in both regression tasks and classification. This technique aggregates multiple decision trees (DTs) through GBM regression trees to build a strong predictive model. Unlike random forests, the decision trees in GBM are not randomized; instead, they are carefully pruned to enhance their performance, a feature derived from the boosting technique^41^. The GBM model iteratively minimizes an objective function, progressively reducing residual errors to achieve the desired accuracy. It uses the negative gradient of the loss function to fit regression trees, continuously refining the prediction performance based on the outcomes of previous weak learners. The implementation of this algorithm is described as follows (Eqs. 5–7).

5 $$\:{G}_{O}=\sum\limits_{i=1}^{N}L({y}_{i},{\upgamma\:})$$

6 $$\:{G}_{m}={G}_{m-1}+arg\:min\sum\limits_{i=1}^{N}\left(L\left({y}_{i},{G}_{m-1}\left({X}_{i}\right)+\:{h}_{m}\left({X}_{i}\right)\right)\right)$$

7 $$\:{\nabla\:}_{\mathrm{L}\mathrm{o}\mathrm{s}\mathrm{s}\:}=-\frac{\partial\:L\left({y}_{i},{G}_{m-1}\left({X}_{i}\right)\right)}{\partial\:{G}_{m-1}\left({X}_{i}\right)}$$

where *h*_*m*_ is the base-function, $\Delta_{Loss}$ is the negative gradient of the loss-function, *x*_*i*_ is the i^th^ independent feature, *G*_*m*_ is the strong-learner, *y*_*i*_ is the actual findings of the *i*^th^ sample, *γ* is the optimal step-length, *L* is the loss-function, and *G*_*O*_ is the weak-learner.

#### Artificial neural networks (ANNs)

The foundational principles of ANNs originate from initial efforts to emulate the sensory processing functions of the human brain^45,46^. An ANN is typically structured with three primary layers: an input layer, one or more intermediate hidden layers, and an output layer, each interconnected via adjustable weight parameters. The computational sequence commences with the input layer acquiring external data, which is subsequently relayed to the hidden layers without initial modification. The hidden neurons execute the core processing tasks by isolating and interpreting significant patterns or features within the data, thereby enabling the network to learn and represent intricate mappings between the input and output variables. Following this, the output layer neurons integrate the aggregated signals from prior layers to generate the network’s ultimate prediction results^45^. Within the framework of an ANN, each neuron initially performs a weighted summation of inputs originating from the preceding layer, incorporating an additional bias term to account for systematic deviations. The associated weights and biases are iteratively refined throughout the training process to enhance model accuracy. Subsequently, the aggregated input is subjected to a nonlinear transformation via activation functions—such as the sigmoid function—within the hidden layers, enabling the network to model intricate and nonlinear data patterns. The detailed architecture and hyperparameters of the developed ANN model are summarized in Table 2. This configuration, which includes two hidden layers, ReLU activation, dropout regularization, L2 regularization, and early stopping, was carefully tuned to achieve high predictive performance while mitigating overfitting risks on the moderate-sized dataset (*n* = 278). The learning efficacy of the ANN is quantitatively evaluated by minimizing a designated loss function, most commonly the root mean square error (RMSE), or by meeting predefined convergence criteria that signal optimization stability.

### SHapley additive exPlanations (SHAP)

Eq. 8 describes the linear combination of input features that the SHAP explanation model can reflect^47^.

8 $$\:\mathrm{g}\left({a}^{{\upalpha\:}}\right)={{\upphi\:}\:}_{o}\:\sum\limits_{i=1}^{N}{{\upphi\:}\:}_{i}{\mathrm{a}\:}_{i}$$

where *a*^*α*^ ∈ {0,1}^*N*^, N represents the total number of input features, $\:{\phi\:\:}_{o}$ corresponds to the model’s baseline output when no features are present, and $\:{\phi\:\:}_{i}\:$denotes the feature attribution value for the $\:{a}_{i}$ feature, providing insight into its contribution to the model’s prediction.

### Hyperparameter optimization

Hyperparameters for all models were systematically tuned using grid search combined with 5-fold cross-validation to maximize the average R² score while minimizing RMSE. The final selected hyperparameters that yielded the best cross-validated performance are summarized in Table 2.

**Table 2:** Optimized hyperparameters for the developed ML models.

Model	Hyperparameter	Searched range	Selected optimal value
L-SVR	Regularization parameter (C)	[0.1, 1, 10, 100, 1000]	100
	Epsilon (ε)	[0.01, 0.1, 0.2, 0.5]	0.1
Poly-SVR	Degree (d)	[2, 3, 4]	3
	Regularization parameter (C)	[0.1, 1, 10, 100]	10
RBF-SVR	Regularization parameter (C)	[0.1, 1, 10, 100, 1000]	100
	Gamma (γ)	[0.001, 0.01, 0.1, 1]	0.01
	Epsilon (ε)	[0.01, 0.1, 0.2]	0.1
GBM	Number of estimators	[100, 200, 300, 500]	300
	Learning rate	[0.01, 0.05, 0.1]	0.05
	Maximum depth	[3, 5, 7]	5
ANN	Hidden layers & neurons	Various	(128, 64)
	Dropout rate	[0.1, 0.2, 0.3]	0.2
	L2 regularization	[0.0001, 0.001, 0.01]	0.001
	Optimizer & Learning rate	Adam	Adam (0.001)
	Early stopping patience	–	50 epochs

### Evaluating ML models: critical performance metrics

To ensure robustness and minimize the effect of random data partitioning, 5-fold cross-validation (k = 5) was implemented on the full dataset in addition to the 75/25 train-test split. In each fold, approximately 222 samples were used for training and 56 for validation. Performance metrics were averaged across all 5 folds^36,48^. The results of the 5-fold CV confirmed the strong predictive capability of the developed models, with low variability across folds, particularly for the top-performing ANN and RBF-SVR models. The performance of the models was subsequently validated and assessed using the testing set. To achieve satisfactory predictive accuracy, hyperparameters within the algorithms were adjusted iteratively. This approach, which focuses on optimizing specific hyperparameters rather than exploring all at once, significantly reduced both computational complexity and time. The disparity between the predicted and actual outcomes was quantified using error values and error percentages, calculated using Eqs. 9 and 10. Additionally, standard statistical metrics, as outlined in Table 3, were employed to provide a comprehensive evaluation of the ML models.

9 $$\:Error={y}_{i}^{{\prime\:}}-{y}_{i}$$

10 $$\:Error\:percentage=\frac{{y}_{i}^{{\prime\:}}-{y}_{i}}{{y}_{i}}$$

Where *y*_*i*_: actual result of the *i*^th^ sample, *yi’*: predictive result of the *i*^th^ sample.

The evaluation metrics employed in this investigation provide insights into the predictive capabilities of the ML models, as demonstrated in Table 3. Elevated coefficient of determination (R²) values signify enhanced predictive accuracy, with measurements approaching unity reflecting a stronger correspondence between observed and predicted outcomes. A Mean Relative Error (MRE) value of 1 similarly represents perfect prediction accuracy. Contrariwise, lower values of error metrics such as Mean Absolute Percentage Error (MAPE), Mean Absolute Error (MAE), Root Mean Square Error (RMSE), and Mean Squared Error (MSE) indicate superior predictive performance.

**Table 3:** Criteria for evaluating the proposed machine learning (ML) approaches.

Establishment of (validation parameters)	Equation	Establishment of (validation parameters)	Equation
R²	$\:1-\frac{{\sum\:}_{i}{\left({y}_{i}-{\widehat{y}}_{i}\right)}^{2}}{{\sum\:}_{i}{\left({y}_{i}-{\stackrel{-}{y}}_{i}\right)}^{2}}$	RMSE	$\:\sqrt{\frac{1}{n}\sum\:_{i=1}^{n}{({y}_{i}-{\widehat{y}}_{i})}^{2}}$
MSE	$\:\frac{1}{n}\sum\:_{i=1}^{n}{({y}_{i}-{\widehat{y}}_{i})}^{2}$	MAE	$\:\frac{1}{n}\sum\:_{i=1}^{n}\left|{y}_{i}-{\widehat{y}}_{i}\right|$

## Results and discussion

### Predictive efficacy and verification

**Fig. 5:**
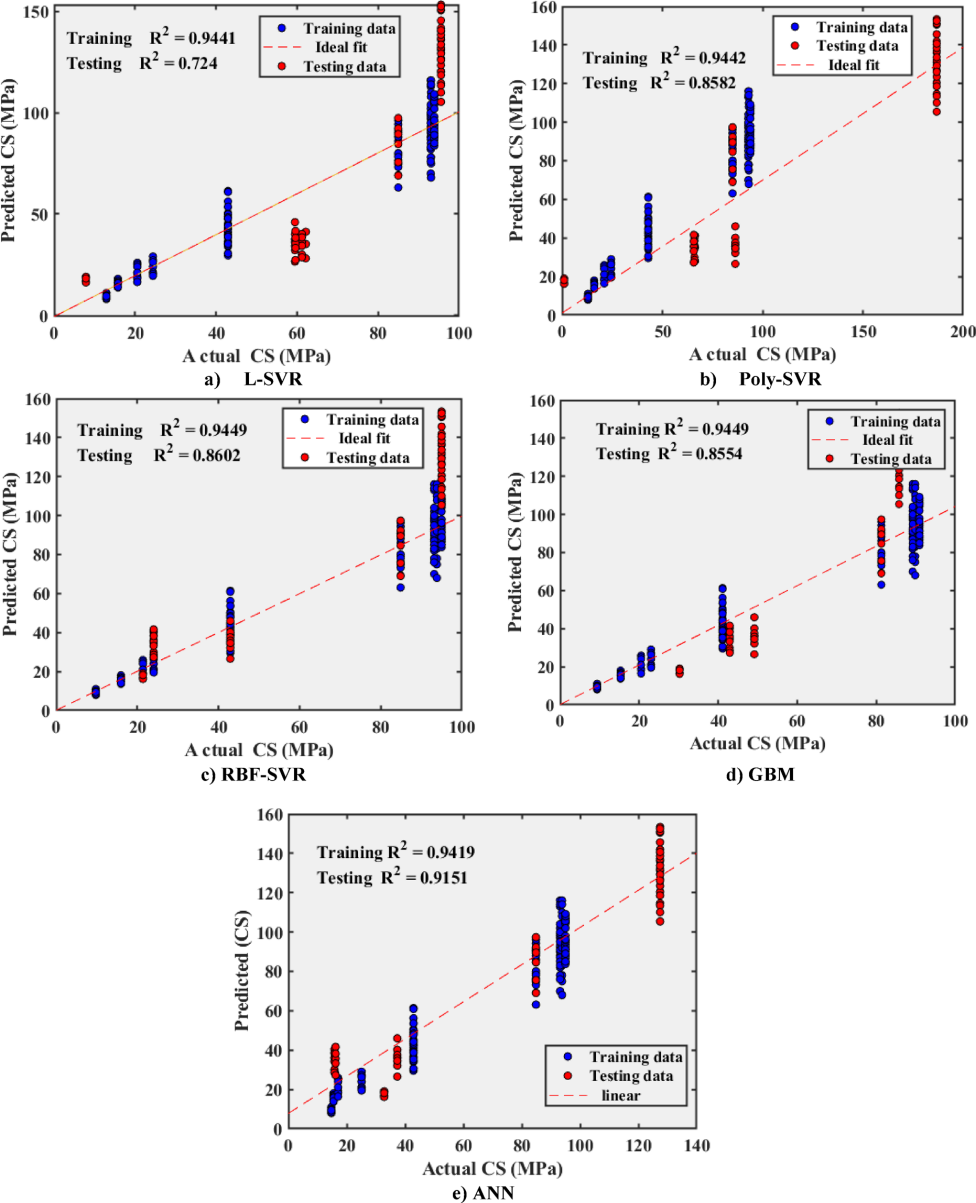
Evaluation of the concordance between measured and forecasted values generated by the proposed models.

Tables 4 and 5 summarize the four-quantitative metrics R², MAE, MSE, and RMSE used to evaluate the efficacy of each in predicting the CS of 3DPFRC for both data partitions for training and testing. Fig. 5a–e depicts a comparative analysis of the actual versus predicted CS values generated by the ML models during the training and validation phases. The degree to which the data points converge along the theoretical 1:1 regression line serves as an indicator of the model’s predictive precision and robustness^49^. The ANN and RBF-SVR models proficiently capture the contribution of model inputs to CS prediction, highlighting their capacity to model the intricate interdependencies among the variables. The ML models exhibit similar levels of accuracy, with R^2^ values of 0.94 across all models in the training dataset, demonstrating a strong correlation between the predicted outputs and the observed values. In the testing dataset, the ANN model reaches a high correlation coefficient of 0.93 for CS prediction. Additionally, RBF-SVR and GBM models achieve R² values of 0.86 and 0.855, respectively, while Poly-SVR models attain 0.85. L-SVR exhibits lower R² values of 0.72 (Fig. 5a). The reported results were further validated using 5-fold cross-validation, which confirmed the stability and robustness of the models with low variability across folds. The model evaluation process analyzed prediction accuracy through the application of both RMSE and MAE performance measurement techniques. These two assessment methods provide an in-depth understanding of predictive precision through quantitative evaluation of prediction differences between expected and observed values. The testing data showed that the ANN model produced an RMSE value of 10.08 MPa and an MAE value of 7.79 MPa, which represents a relatively low prediction error. The RBF-SVR model had higher prediction errors compared to the ANN model because its RMSE value reached 14.47 MPa, and its MAE value was 9.31 MPa during the CS prediction evaluation. The comparison between the models shows that the ANN model demonstrates significantly better performance based on reduced error levels (Fig. 5c and e). The findings indicate that the ANN model makes more accurate predictions while the RBF-SVR model needs further adjustments to achieve similar performance levels.

**Table 4:** Evaluation of ML models’ performance (training stage).

Model	*R*^2^-value	RMSE (MPa)	MSE (MPa²)	MAE (MPa)
L-SVR	0.94	8.19	67.11	6.07
Poly-SVR	0.94	8.19	67.15	6.09
RBF-SVR	0.94	8.19	67.11	6.07
GBM	0.94	8.64	74.72	6.30
ANN	0.94	8.32	69.23	6.36

**Table 5:** Evaluation of ML models’ performance using statistical parameters (testing stage).

Model	*R*^2^-value	RMSE (MPa)	MSE (MPa²)	MAE (MPa)
L-SVR	0.72	22.6	512.74	14.05
Poly-SVR	0.83	15.9	255.67	10.86
RBF-SVR	0.86	14.47	209.56	9.31
GBM	0.85	17.08	291.66	10.58
ANN	0.93	10.08	101.73	7.79

**Fig. 6:**
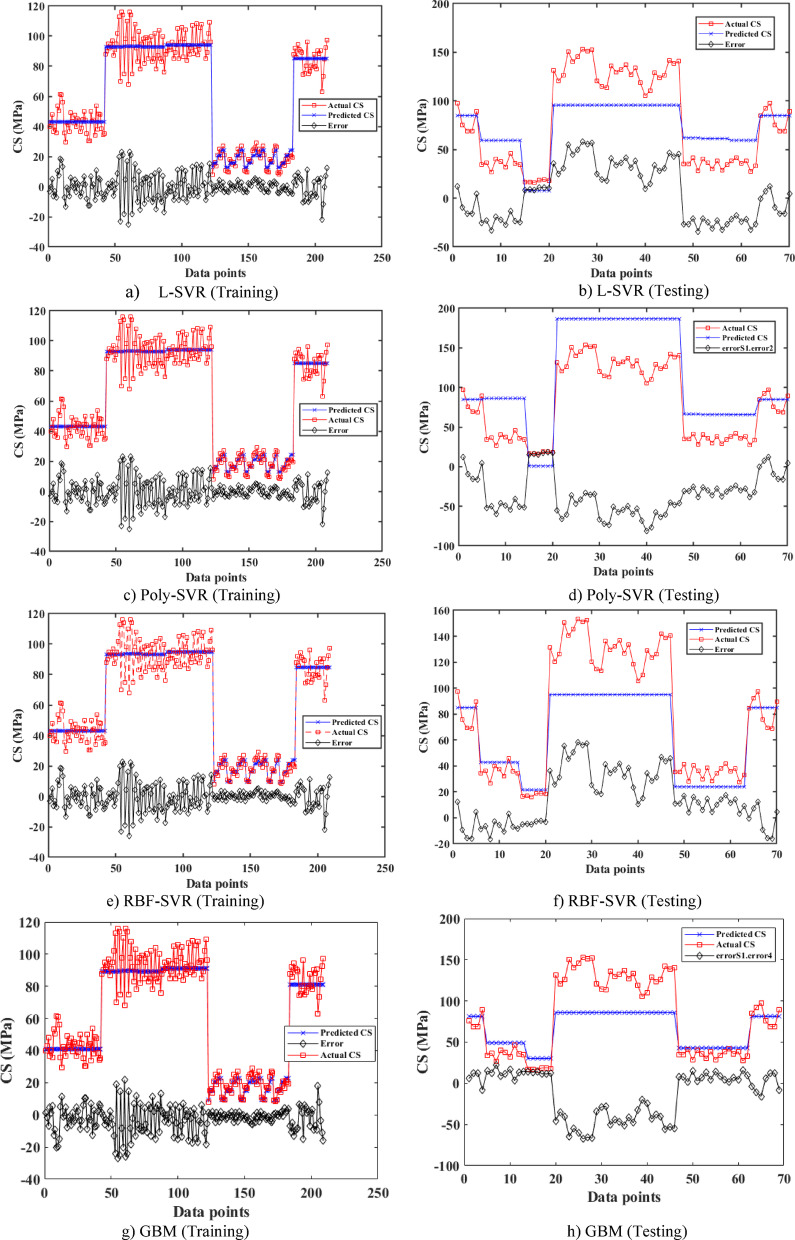

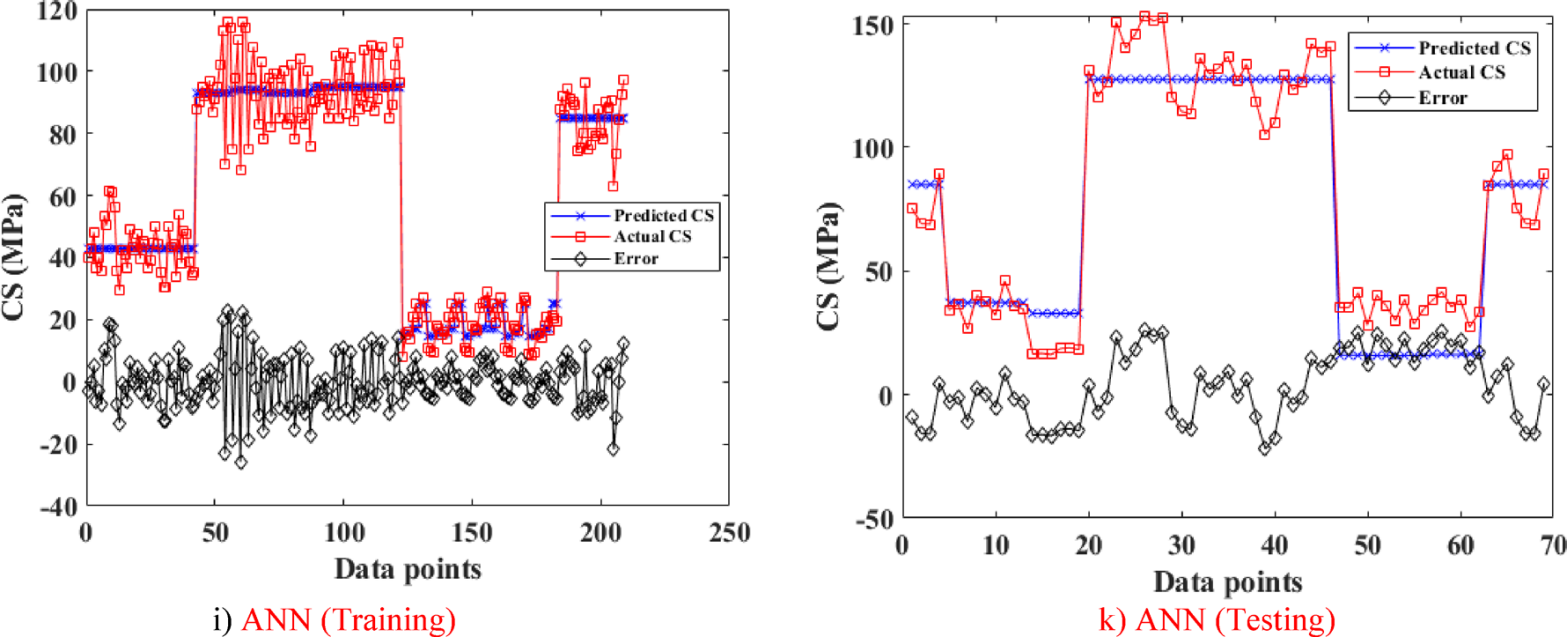
Analysis of real compressive strength (CS) values and corresponding prediction errors from the models.

Fig. 6a–k presents the comparative evaluation of the ML models’ predictive accuracy for CS on both the training and validation datasets. ANN and RBF-SVR models demonstrate a closer alignment with the experimental datasets in comparison to L-SVR, Poly-SVR, and GBM throughout the training and testing stages. However, a closer comparison of the testing dataset in both figures indicates that ANN tracks the experimental datasets more accurately. Additionally, Fig. 6a–k presents the distribution of errors, with boxplots comparing the performance of each ML model. These figures demonstrate that the predictions align closely with the experimental results, maintaining a low error margin. Almost all of the samples, more than 95% of the samples, fall within the ± 10% error range for the ANN model, and approximately 85–90% for the RBF-SVR model in the testing set. In contrast, other models exhibit a higher error margin when predicting CS for normal, HPC, and UHPC 3DPFRC. For instance, during the testing phase, the error values for the L-SVR model exceed 50 MPa, highlighting its poor prediction performance in comparison with ANN and RFB-SVR models.

**Fig. 7:**
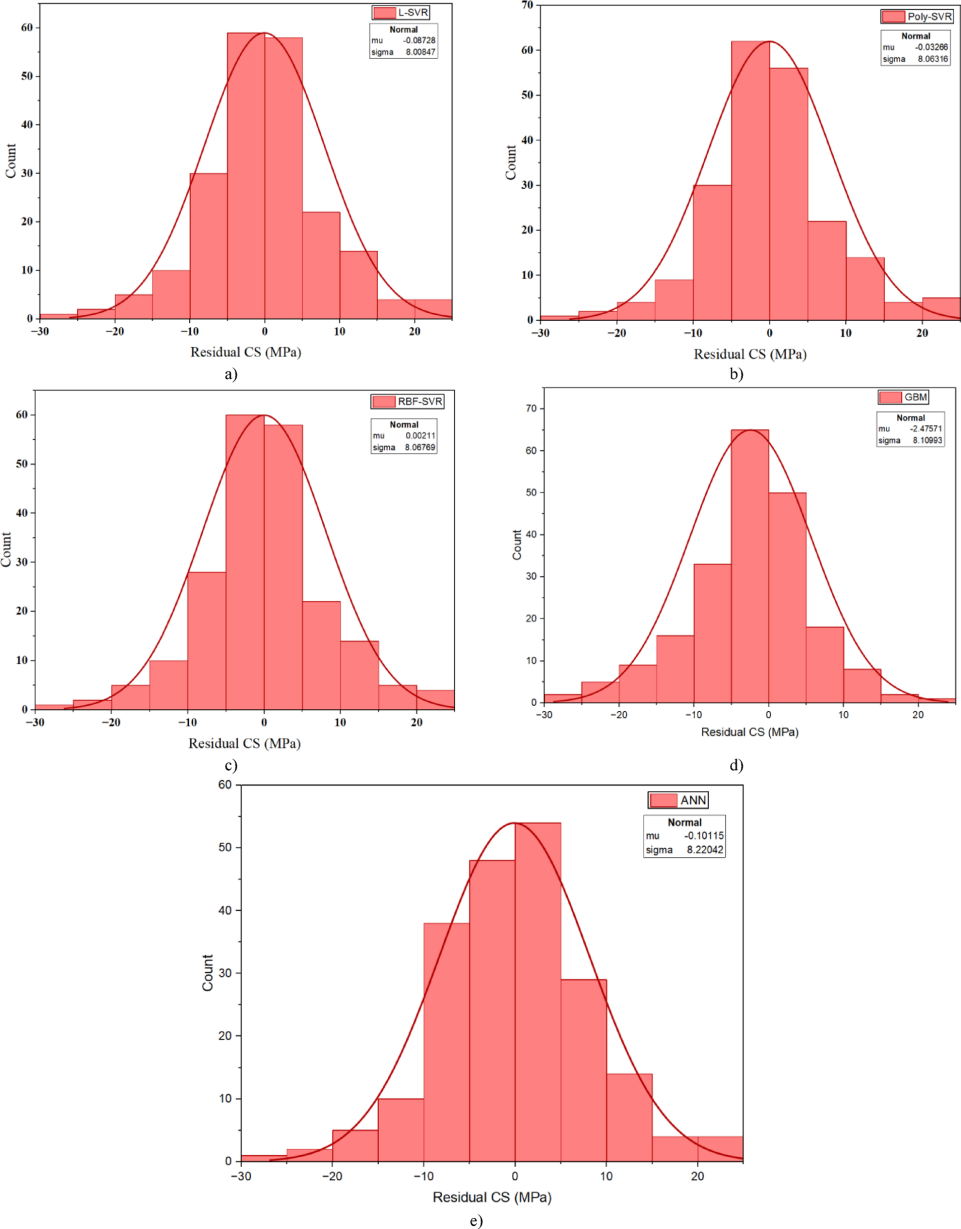
**(a–e)** Residuals for CS (training phase).

**Fig. 8:**
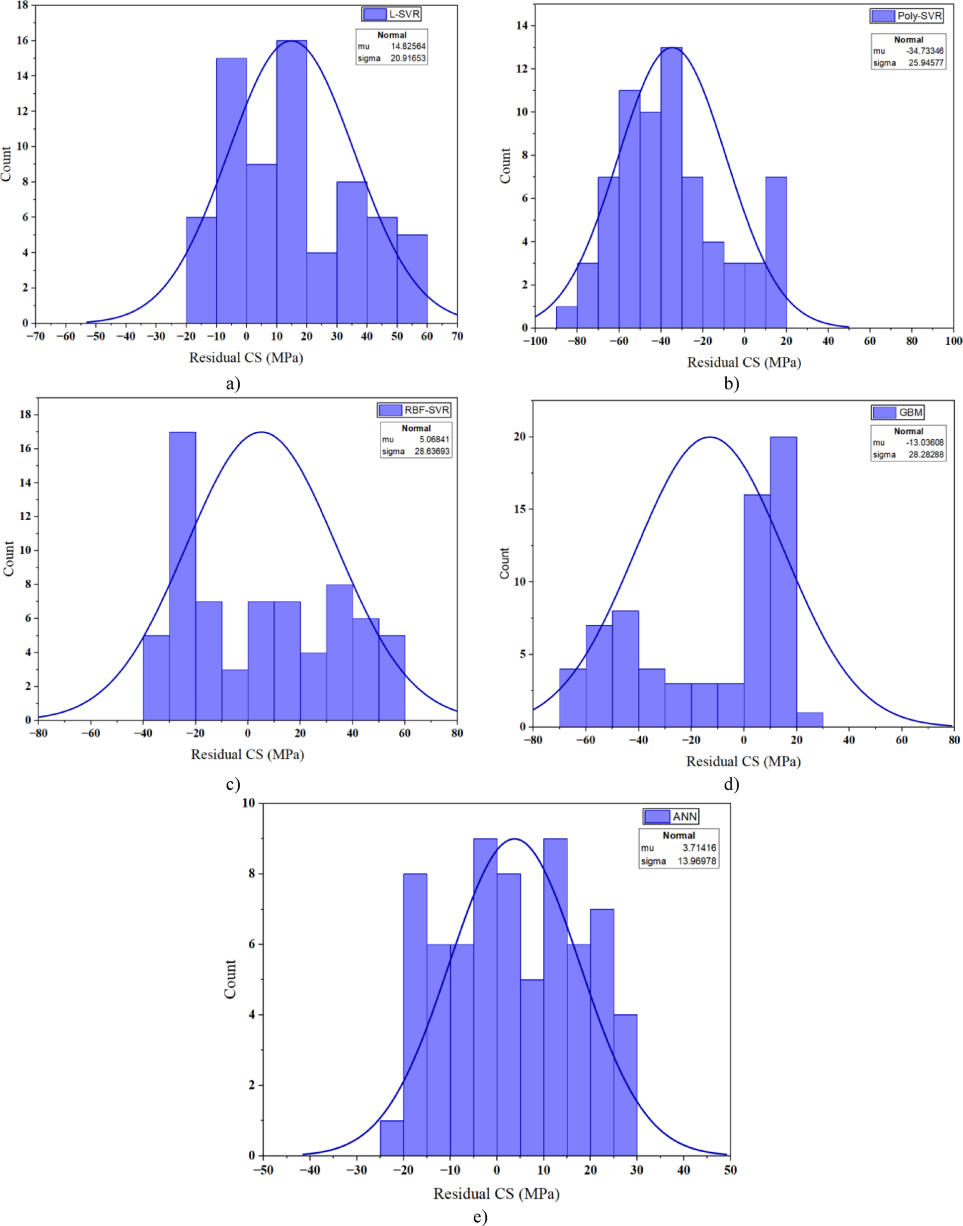
**(a–e)** Residuals for compressive strength (CS) (testing phase).

Figs. 7a–e and 8a–e also display the residual values for all ML models, which confirm similar trends in error distribution for the ML models. The results indicate that ANN and RBF-SVR models exhibit a normal error distribution compared to L-SVR, Poly-SVR, and GBM. Among these, ANN demonstrates a high confidence level and a narrow prediction range for the CS of 3DPFRC. Generally, ANN and RBF-SVR produce the most robust results with minimal uncertainty, followed by RBF-SVR, Poly-SVR, GBM, and L-SVR, respectively.

### A comparative analysis of the models

**Fig. 9:**
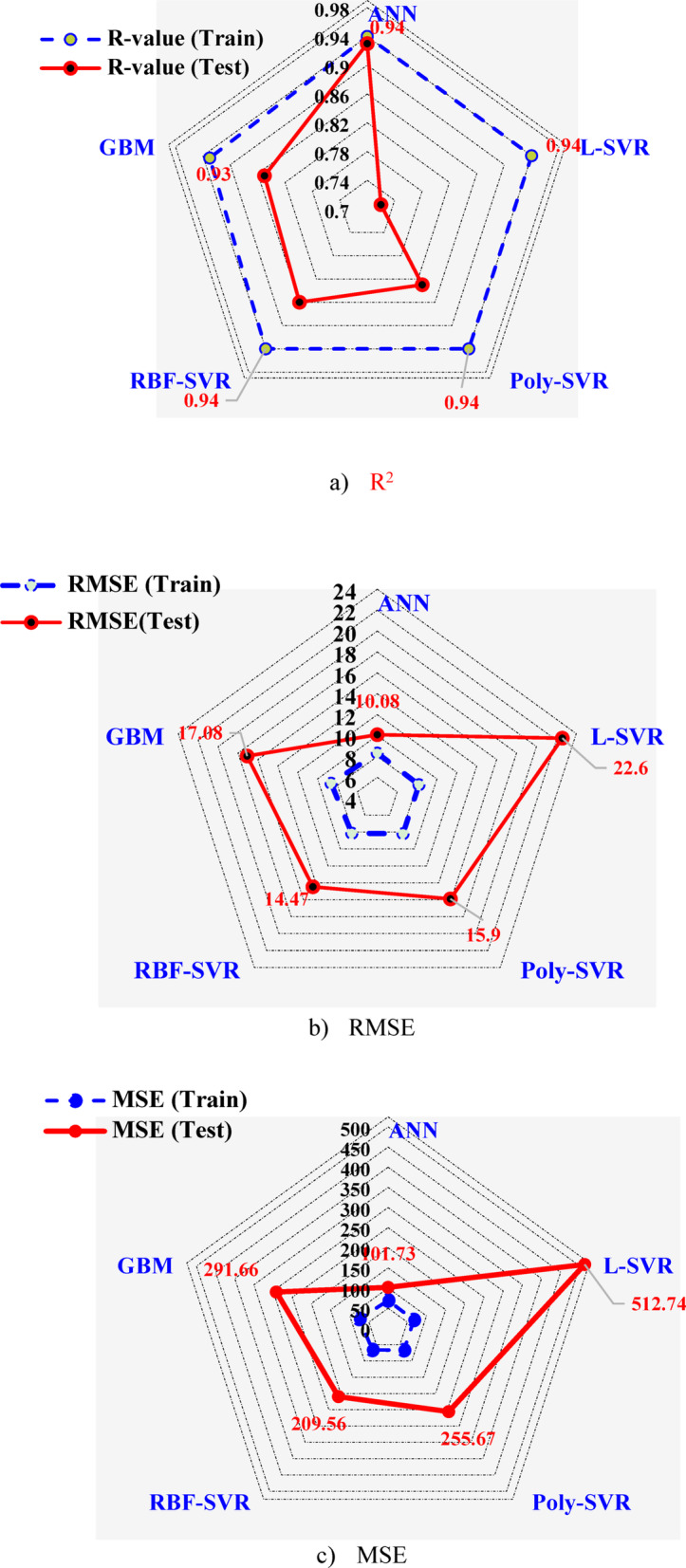

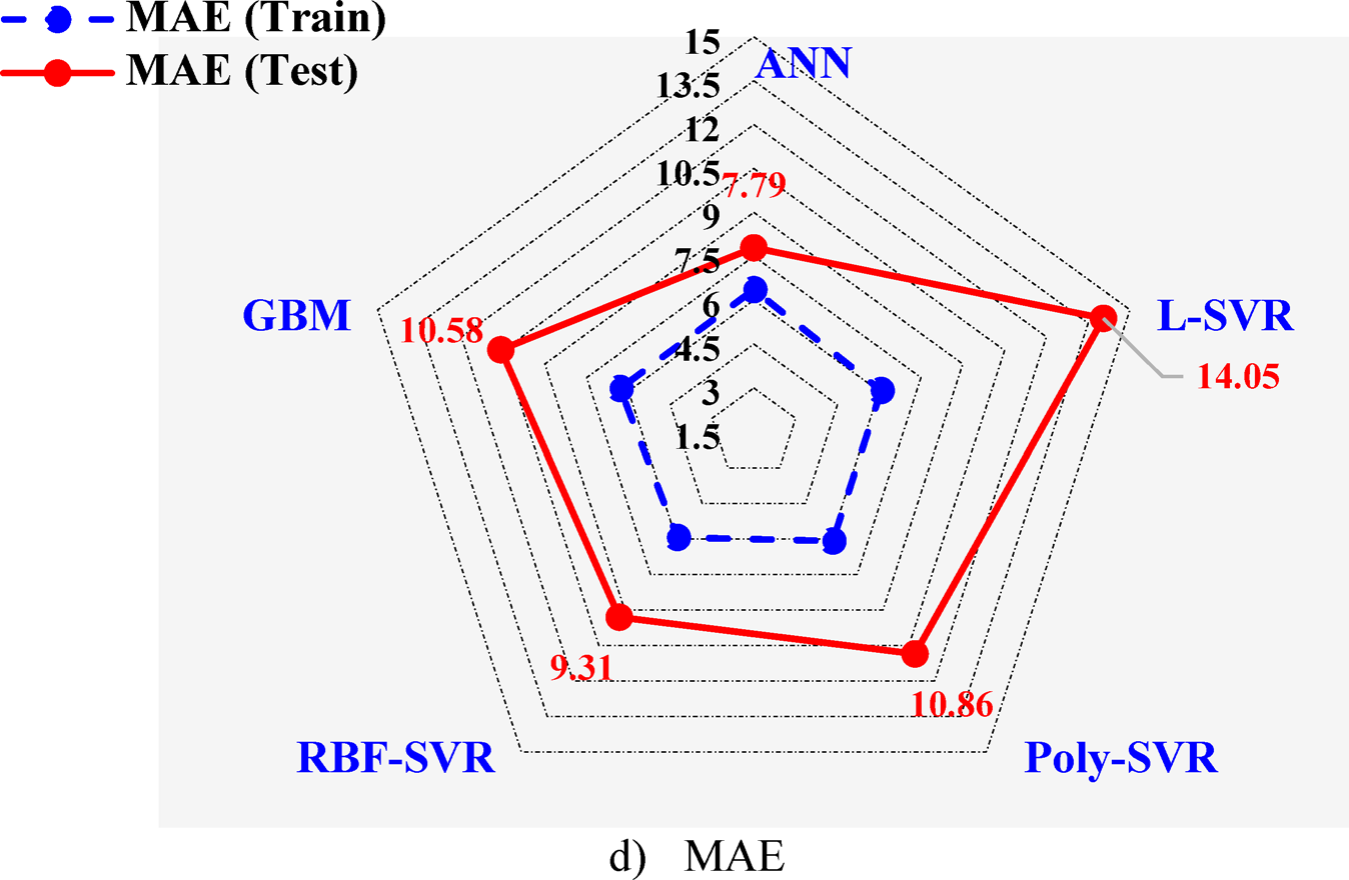
**(a–d)** Comparison of the proposed machine learning (ML) models.

Fig. 9 presents a radar plot comparing various developed models, which is particularly effective for visualizing multivariate data. Each of the four ML models tested in the study is represented by a spoke radiating from a central point, with the models arranged in a radar plot format. In terms of correlation between actual and predicted results, the ANN model demonstrated superior performance during testing in CS prediction, achieving an R^2^ of 0.92. In the testing phase, the RBF-SVR model exhibited a robust correlation, attaining an R^2^ of 0.86, while the prediction of CS for other L-SVR and Poly-SVR achieving 0.72 and 0.83. The GBM model also exhibited comparable correlation results in CS prediction. During evaluation on the testing dataset, the L-SVR model yielded the highest errors, with an RMSE of 22.6 MPa, a MSE of 512.74 MPa, and a MAE of 14.05 MPa. Conversely, the RBF-SVR model outperformed L-SVR by achieving reduced error metrics, registering a RMSE of 14.47 MPa, MSE of 209.56 MPa, and MAE of 9.31 MPa.

Additionally, the 5-fold cross-validation results demonstrate acceptable stability across all models. The ANN model exhibited the best average performance and lowest variability, followed by RBF-SVR, as presented in Table 6. The larger standard deviation observed for L-SVR indicates higher sensitivity to data partitioning, consistent with its weaker performance on the independent test set.

**Table 6:** Performance metrics from 5-fold cross-validation (Mean ± Standard Deviation).

Model	*R*² (Mean ± SD)	RMSE (MPa)	MAE (MPa)
L-SVR	0.79 ± 0.07	19.8 ± 3.1	13.2 ± 1.9
Poly-SVR	0.86 ± 0.05	16.1 ± 2.4	10.9 ± 1.6
RBF-SVR	0.88 ± 0.03	14.6 ± 1.9	9.5 ± 1.2
GBM	0.87 ± 0.04	15.3 ± 2.2	10.1 ± 1.4
ANN	0.91 ± 0.02	12.1 ± 1.6	8.4 ± 1.1

The present study distinguishes itself by developing and comparing several ML models, including different SVR variants (*L-SVR*,* Poly-SVR*,* and RBF-SVR*), as well as GBM and ANN models, using a comprehensive dataset of 278 samples that covers normal-, high-, and ultra-high-performance 3DPFRC mixtures. Among the developed models, the ANN model achieved the highest prediction accuracy during testing, while the RBF-SVR model demonstrated a strong balance between prediction capability, simplicity, and computational efficiency. Compared to previous studies, the current work offers several advantages. While Alyami et al.^24^ achieved high accuracy (98.7%) using decision trees on 299 samples, their model lacks the diversity of SVR kernels explored here. Iqbal et al.^50^ reported a slightly higher R² of 0.95 with CNN, but the current ANN model (R² = 0.93) remains highly competitive while using a simpler architecture, as presented in Table 7. Rehman et al.^51^ reached R² up to 0.982 with XGBoost enhanced by data augmentation; however, the present study achieved strong performance (R² = 0.93) without relying on synthetic data generation. Albostami et al.^52^ obtained R² = 0.92 using Gradient Boosting, which is comparable to the current GBM (R² = 0.85), but the current ANN outperformed it. Notably, the current study provides a more balanced evaluation by testing multiple SVR variants with RBF-SVR, showing robust results (R² = 0.86). In contrast to studies with smaller datasets, such as Izadgoshasb et al.^53^(65 samples), or those focused mainly on boosting algorithms^51,52^, or interpretable models only^54^, the present research combines a relatively large and diverse dataset, multiple modeling approaches, and detailed SHAP interpretability focused on key mix design parameters. This makes the developed models particularly practical for real-world 3D printing applications^55^.

**Table 7:** Comparative evaluation with recent studies for predicting CS of 3DPFRC.

Ref.	Dataset	Best model(s)	Key performance (testing/best)	Notes
Current study	3DPFRC (Normal, HPC, and UHPC), 278 samples	ANN (RBF-SVR competitive)	R² = 0.93 (ANN), 0.86 (RBF-SVR)	Largest dataset; multiple SVR kernels and SHAP analysis
^24^	3D-printed FRC, 299 samples	DT	Accuracy = 98.7% (high R² implied)	DT outperformed SVR, RF, GB, GEP, etc.
^50^	Anisotropic 3D printed concrete, 200 samples.	Multiple neural networks	R² = 0.95 (testing)	Strong neural network performance.
^51^	3DPC (anisotropic CS and slump), 1679 data points with 23 features	XGBoost (with data augmentation)	R² up to 0.982 (cast), = 0.95 (printed)	Data augmentation significantly improved performance
^52^	3DP-FRC	Gradient Boosting (GB)	R² = 0.92	GEP is also strong; SHAP used
^56^	3DPC, 77 mix designs	SVM and XGBoost	R² = 0.89	Focused on anisotropic properties
^53^	3D-printed mortar, = 65 samples	ANN and MOGOA	Correlation = 0.96	Hybrid optimization for small dataset
^54^	3DP-FRC, 299 samples	Interpretable machine learning algorithms	Strong performance (details vary)	Interpretable models

DT: Decision tree, GEP: Gene expression programming, MOGOA: Multi-objective grasshopper optimization algorithm.

### Analysis of model explanations provided by the ML model

Although advanced ML models exhibit strong predictive capabilities for the CS of 3DPFRC, the increasing complexity of these models necessitates further clarification of their results. On the other hand, ML models not only provide enhanced performance but also offer built-in interpretability, which will be comprehensively examined in this sections ^37,39^.

#### Global explanation

**Fig. 10:**
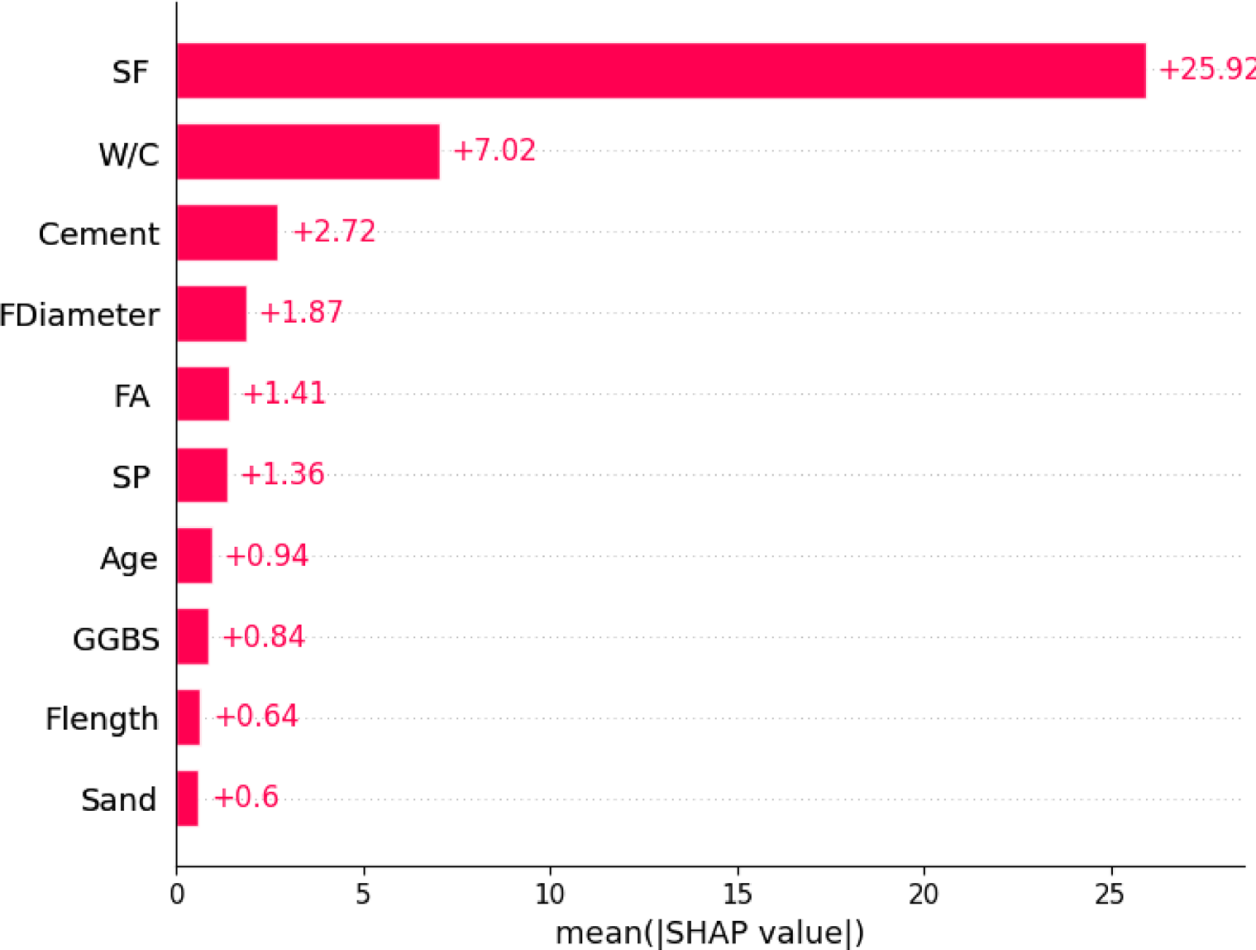
Feature importance determined using the mean Shapley additive explanation (SHAP) values.

The global explanations provided by ML models enable the assessment of the influence of specific input variables based on the prediction outcomes, offering insights into the model’s rationality based on prior knowledge. The feature importance, illustrated in Fig. 10, is calculated from each feature’s contribution to the predicted CS within the ANN model. By applying the feature’s value to the corresponding shape function, its direct contribution can be determined. The absolute contributions across all training samples are averaged to derive the feature importance, reflecting the overall influence on CS. The SHAP values are ranked from highest to lowest based on their effect, identifying the most influential input parameters on CS. These factors are critical in determining the strength of 3DPFRC, which is closely tied to CS ^47,57,58^. SF emerged as the most influential factor, significantly improving CS, with the W/C ratio and cement content following in importance, which also played pivotal roles, while curing age, fiber diameter, and SP were found to have lesser effects, as presented in Fig. 10.

**Fig. 11:**
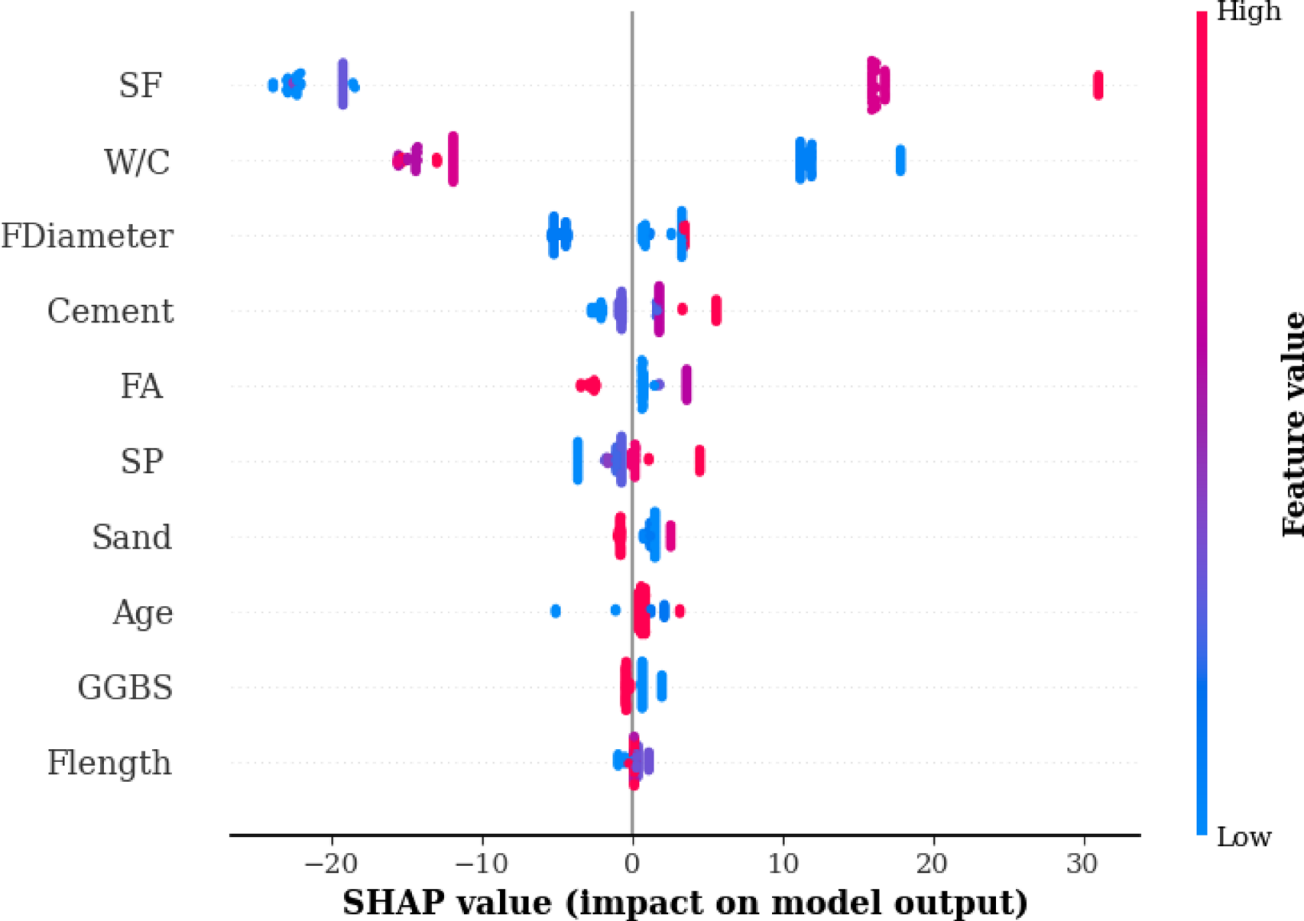
Shapley additive explanation (SHAP) summary plot highlighting feature impact on compressive strength (CS).

To clarify the nature of these associations, a SHAP summary plot is provided in Fig. 11, which visually demonstrates the correlation direction between the input parameters and CS. In this visualization, the input features are ordered along the vertical axis according to their relative importance, whereas the horizontal axis quantifies the associated SHAP values. The color of the dots transitions from blue (signifying weak intensity) to red (signifying strong intensity), corresponding to the values of the data points. The x-axis shows how variations in the intensity of each input feature correspond to the magnitude of the output predictions, helping to clarify whether an increase or decrease in feature value leads to higher or lower CS predictions. Notably, a higher SF content and lower W/C ratio exhibit a positive SHAP value (approximately + 25), while a higher W/C ratio shows a negative SHAP value (around − 25), indicating that a higher SF and lower W/C ratio enhances CS, and vice versa. A similar trend is observed for F-diameter in Fig. 11, which highlights the feature impact using the SHAP summary plot. It is observed that the SF, W/C ratio, and fiber diameters are the highest parameters. Specifically, Pham et al. ^4^ performed an analysis of the influence of short, straight steel fibers ranging in length from 3 mm to 6 mm, with a diameter of 0.2 μm, on the mechanical performance of 3DPFRC. Their findings revealed that incorporating steel fibers enhanced the CS of printed specimens by 24–26%. Incorporating 0.75-1 vol% of 6 mm steel fibers into the printed concrete significantly improved its bending capabilities, particularly in the printing direction. Additionally, features such as FA, SP, cement, and curing age show positive influences on CS, as they trend toward the right side of the plot. Conversely, increasing sand content significantly reduces CS, as indicated by its negative SHAP values.

**Fig. 12:**
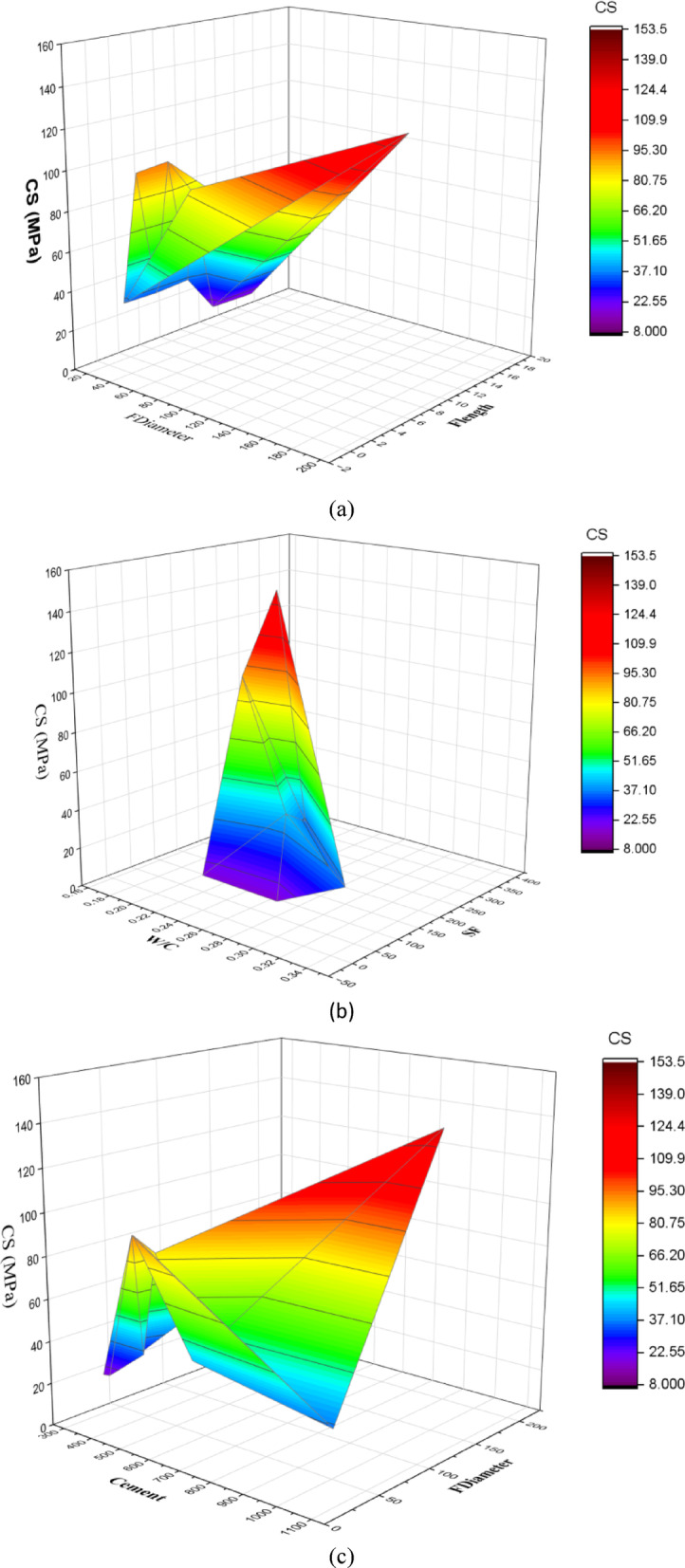
Effect of the two input parameters on the compressive strength (CS) during the artificial neural networks (ANN) model prediction: (**a**) fiber diameter and length, (**b**) SF and water-to-cement ratio (W/C ratio), and (**c**) cement and fiber diameter.

Fig. 12 shows the effects of two input parameters on 3DPFRC CS while predicting with the ANN model. In Fig. 12a, we see the relationship between fiber diameter and fiber length, where higher CS values correlating to optimal fiber diameter and length parameters. In Fig. 12b, we report the effects of SF and W/C ratio. Increasing the amount of SF and maintaining an optimal W/C ratio would significantly increase CS values. Finally, in Fig. 12c, we see the interaction between cement content and fiber diameter, where an optimal result occurred at the highest fiber diameter with a cement content of 900 kg/m^3^. These 3D surface plots, which were confirmed by a high R² value of 0.93 for the ANN model on the test set, demonstrate the importance of these parameters for improving the mechanical performance of 3DPFRC.

#### Local interpretation

**Fig. 13:**
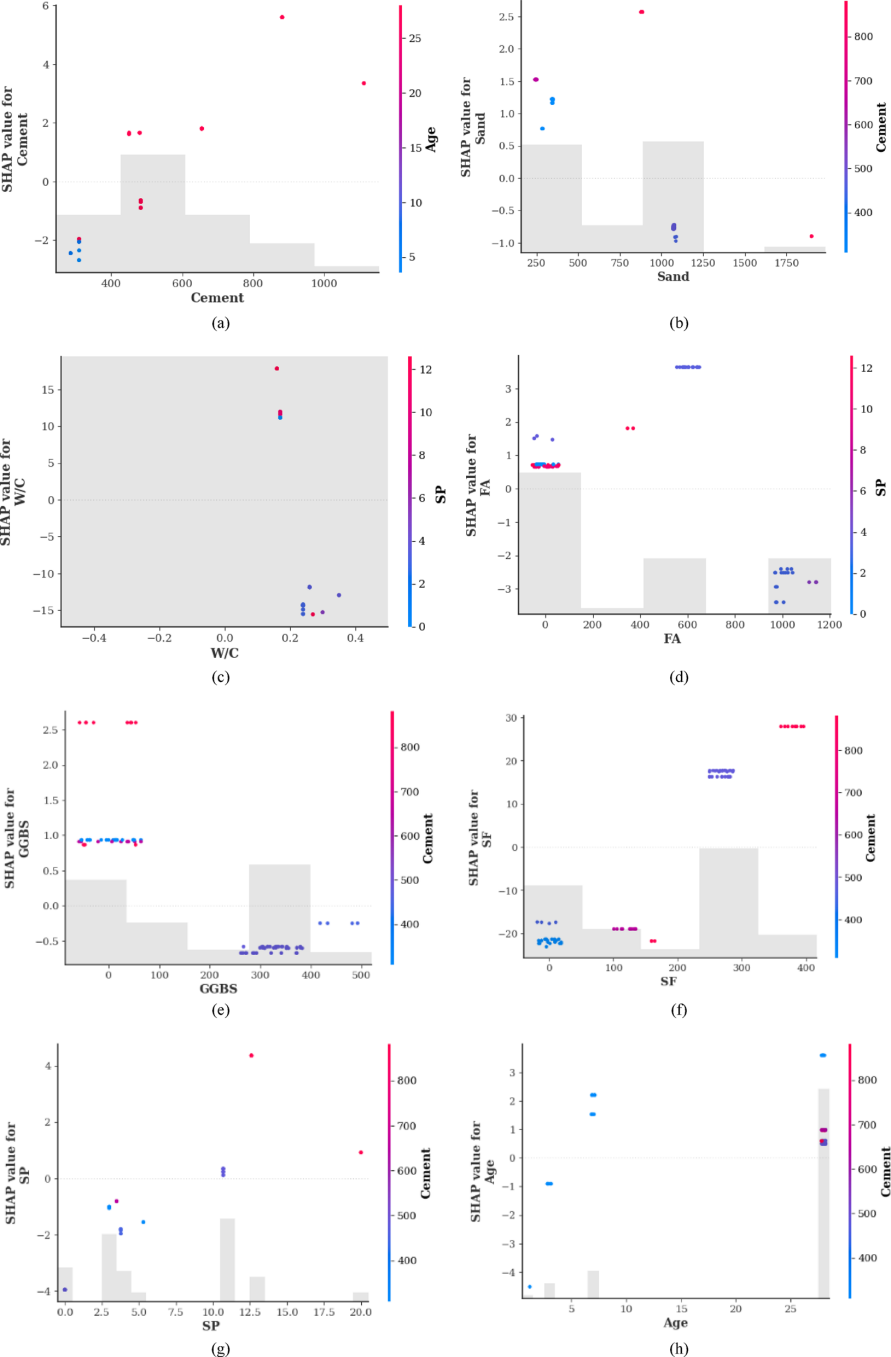

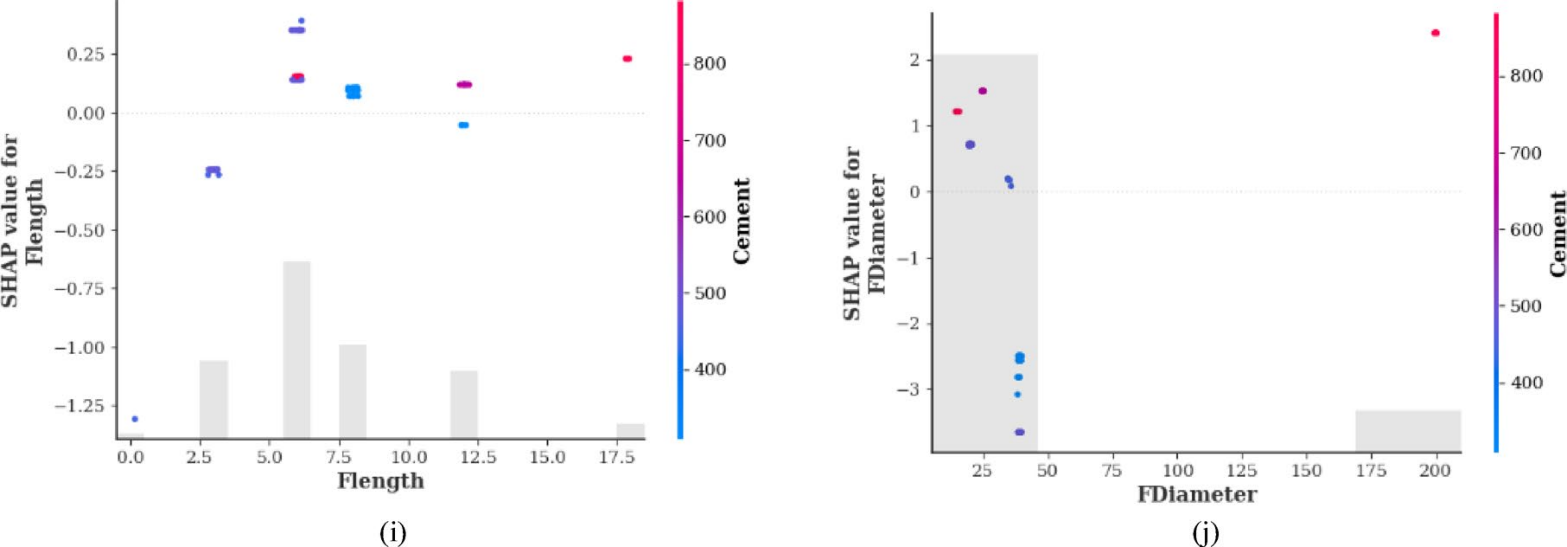
Shapley additive explanation (SHAP) plots: (**a**) cement, (**b**) sand, (**c**) water-to-cement ratio (W/C ratio), (**d**) fly ash (FA), (**e**) ground granulated blast-furnace slag (GGBS), (**f**) silica fume (SF), (**g**) superplasticizer (SP), (**h**) curing age, (**i**) fiber length (F-length), and (**j**) fiber diameter (F-Diameter).

It is important to note that SHAP analysis explains how the trained models utilize the input features to generate predictions and does not establish direct physical causation. The observed feature importance and effects should be interpreted as statistical associations learned by the models, which are then compared with existing experimental evidence from the literature. Local SHAP interpretations serve a critical function in the optimization of input parameters, whereas global SHAP explanations offer an in-depth insight into the importance and directional impact of input features. Fig. 13a–j presents the SHAP dependence charts corresponding to the input variables under analysis. As shown in Fig. 13a, higher cement content is associated with increased CS, while lower sand content also contributes positively to CS (Fig. 13b). Numerous studies have explored the impact of cement content and sand on the CS of concrete and 3DPFRC ^13,59–62^. An increase in cement content generally enhances CS by improving the binder matrix and reducing voids. Conversely, excessive sand content can reduce workability and lead to segregation, negatively impacting strength. Thus, Nguyen et al. ^57^ reported that substituting 30% of silica sand with ground silica improved the CS by up to 15%, attributed to the reduced porosity and improved particle packing. Additionally, Fig. 13c shows the effect of the W/C ratio on CS development. It was observed that lower W/C ratios usually lead to improved CS by reducing porosity and increasing matrix density. Wang et al. ^4^ highlight the efficiency of additive manufacturing for creating intricate concrete elements without support structures, employing selective binder activation in particle-bed printing. The optimal parameters were established, consisting of a sand fineness modulus of 2.751, a W/C ratio of 0.151, and a sand-to-cement ratio of 2.0. These attributes contributed to enhanced mechanical strength and dimensional precision, thereby advancing the development of 3D printing methodologies for structural concrete design.

Correspondingly, Figs. 13d to f show notable effects of GGBS, SF, and FA on CS. There exists an optimal range of GGBS content where a balance between porosity and strength can be achieved. The CS of 3DPFRC varies significantly between normal concrete and UHPC. Normal performance concrete typically exhibits CSs ranging from 20 to 40 MPa (Fig. 13a), suitable for conventional structural applications. In contrast, UHPC achieves much higher CSs, often exceeding 120 MPa (Figs. 13b–f), representing an improvement of over 200% compared to the upper range of normal concrete. This significant enhancement is attributed to UHPC’s optimized mix designs, including reduced W/C ratios, high cementitious content, and advanced additives such as SF and SPs ^5,22,30,59,63^. By reducing the water content while maintaining adequate rheological properties, SPs contribute to denser matrix structures, leading to higher CS, as presented in Fig. 13g. Furthermore, Wang et al. ^64^ demonstrated that incorporating SPs improved the dispersion of fibers within the cement matrix, promoting uniform stress distribution and increasing CS. However, excessive use of SPs can lead to segregation and negatively impact structural integrity, as noted by Chen et al. ^65^. Consequently, it is crucial to optimize the SP dosage to attain an optimal balance between workability and CS in 3DPFRC. These outcomes emphasize the pivotal role of SPs in the design of high-performance 3DPFRC formulations. Finally, Fig. 13g and h confirms that CS increases with fiber length and is also affected by the fiber diameter. Additionally, the use of fibers in UHPC further improves strength by enhancing crack resistance and interlayer bonding, making it ideal for highly demanding applications like bridge components and structural retrofitting ^46^. These observations highlight the intricate role of process parameters in influencing the CS of 3DPFRC.

**Fig. 14:**
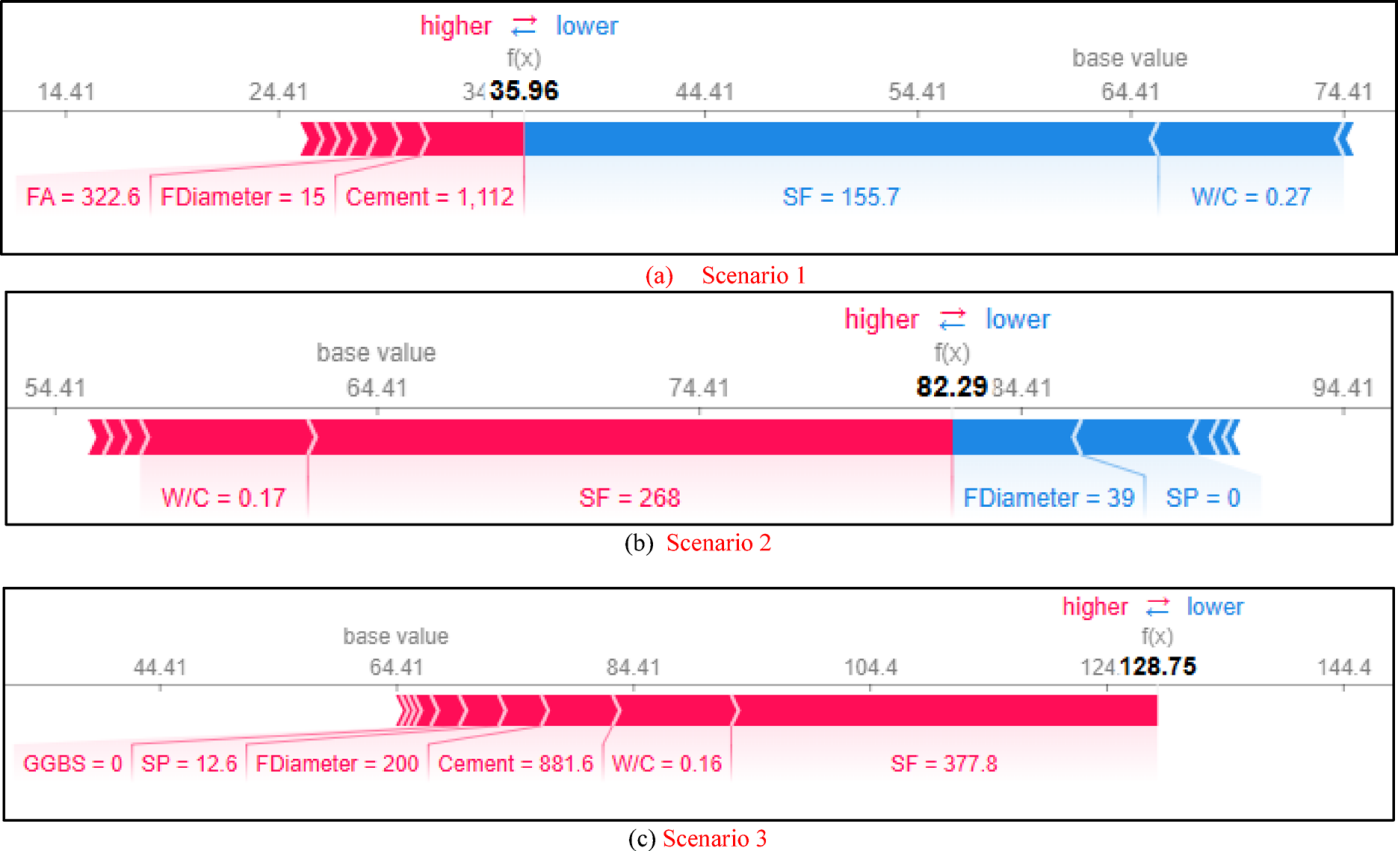
SHAP force plots illustrating local interpretations for selected samples.

Fig. 14a–c demonstrates local explanations by examining specific predictions through SHAP force plots. In this case, lower values of SF and higher values of W/C ratio are associated with a reduction in CS (*Scenario 1*). In Scenario 1, lower SF content and higher W/C ratio are associated with a reduction in predicted CS. In contrast, Scenarios 2 and 3 show that higher SF content and fiber diameter positively contribute to the model’s predictions of CS, while low SP content exerts a negative influence. The emphasized figure, obtained by summing the base value and the associated SHAP values, represents the output prediction generated by the ML algorithm. These outcomes highlight the effectiveness of SHAP analysis in revealing the precise impact of each feature on model predictions, enabling an in-depth understanding of model behavior from both global and local viewpoints ^66^.

## Conclusions, limitations, and perspectives

The present study explored the application of SVR, including L-SVR, Poly-SVR, and RBF-SVR, for evaluating the CS of normal, high, and ultra-high performance 3DPFRC. Additionally, the study conducted a comparative assessment of the SVR models with GBM and ANN models to systematically evaluate their predictive accuracy, computational efficiency, and robustness in forecasting CS. To interpret the models, SHAP was employed. The principal results and inferences drawn from this research are presented below.

1. Among the ML models, the ANN model displayed the utmost precision for CS prediction, achieving an R² value of 0.94 for training and 0.93 for testing. RBF-SVR showed competitive performance with testing R² values of 0.86, surpassing GBM (0.855) and Poly-SVR (0.83) models. L-SVR underperformed with a significantly lower R² value of 0.72 during testing.
2. ANN exhibited the lowest error metrics with RMSE and MAE values of 10.08 MPa and 7.79 MPa, respectively, during testing. Comparatively, RBF-SVR had higher RMSE and MAE values of 14.47 MPa and 10.58 MPa, whereas L-SVR recorded the highest errors of 22.6 MPa (RMSE) and 14.05 MPa (MAE), demonstrating its poor predictive capability.
3. ANN and RBF-SVR models effectively captured the influence of input features on CS, with more than 95% of the samples falling within the ± 10% error range for the ANN model and approximately 85–90% for the RBF-SVR model in the testing set. Other models exhibited higher error margins.
4. SHAP force plots demonstrated specific feature interactions. For example, higher SF content positively impacted CS by + 25 SHAP values, while a higher W/C ratio negatively influenced CS by approximately − 25 SHAP values. Similarly, scenarios indicated that low SP content could reduce CS by 10–15%, emphasizing the need for optimal additive dosages.
5. For achieving high compressive strength (> 80 MPa) while maintaining printability, engineers should target a water-to-cement ratio of 0.18–0.22, silica fume content of 10–15% by weight of cement, cement content of 550–900 kg/m³, and steel fibers with diameter 80–150 μm and length 6–12 mm. These ranges were identified as the most influential parameters through SHAP analysis.
6. The high-performing ANN and RBF-SVR models can be integrated into digital mix design tools or Building Information Modeling (BIM) platforms for real-time prediction and optimization. This approach can significantly reduce the number of costly experimental trials during the development of new 3DPFRC mixes.
7. The present study compiled a dataset of 278 samples from multiple independent published studies, which provides reasonable diversity in mix designs, fiber types, supplementary materials, and testing conditions. The models were rigorously evaluated using a 75/25 train-test split and 5-fold cross-validation. However, we acknowledge two main limitations. First, all data were sourced from the existing literature, with no independent experimental dataset used for external validation. Second, several important printing process parameters (such as printing speed, extrusion pressure, layer interval time, and build orientation) were not included as input features due to inconsistent reporting across the collected studies. These remain common limitations in data-driven research on 3DPC. Future experimental campaigns are planned to generate new independent datasets that incorporate these process parameters, enabling more comprehensive external validation of the developed models, particularly the high-performing ANN and RBF-SVR models.

## Recommendations for future work

The recommendations include, but are not limited to:

1. To enhance the accuracy of CS predictions, additional input variables, including environmental conditions, curing duration, and aggregate characteristics, should be systematically integrated into the analysis.
2. The implementation of predictive models in real-time applications, such as construction monitoring systems, is essential for validating their practical effectiveness and applicability in dynamic operational settings.
3. Investigating advanced hybrid ML frameworks and deep learning methodologies offers the potential to enhance prediction accuracy, surpassing the capabilities of conventional models such as ANN and RBF-SVR.

In future work, the research team plans to perform additional laboratory experiments to generate an independent external validation dataset. This will allow a more comprehensive assessment of the real-world generalizability of the proposed models beyond the current literature-based dataset.

## Supplementary Information

Below is the link to the electronic supplementary material.

Supplementary Material 1

## Data Availability

The authors declare that the data supporting the findings of this study are available within the paper. Should any raw data files be needed in another format, they are available from the corresponding author upon reasonable request.
