# Supplementary material for: Machine learning prediction of compressive strength in 3d printed fiber reinforced concrete using support vector regression and artificial neural networks with shapley additive explanations
Source: Sci Rep. 2026 Jun 25;16:19602. doi: 10.1038/s41598-026-58697-3 (PMC13303825; doi:10.1038/s41598-026-58697-3)
Supplement: Supplementary file 1 — Supplementary Material 1 [file 41598_2026_58697_MOESM1_ESM.docx]

**Supplementary material of the article, titled: “Machine learning prediction of compressive strength in 3d printed fiber reinforced concrete using support vector regression and artificial neural networks with shapley additive explanations”**

**Table S1.** The dataset used to carry out the current study.

| **Cement** | **Sand** | **W/C** | **FA** | **GGBS** | **SF** | **SP** | **Age** | **FDiameter** | **Flength** | **CS** |
| --- | --- | --- | --- | --- | --- | --- | --- | --- | --- | --- |
| 655 | 246 | 0.26 | 604 | 0 | 118 | 3.5 | 28 | 25 | 12 | 39.82855 |
| 655 | 246 | 0.26 | 604 | 0 | 118 | 3.5 | 28 | 25 | 12 | 42.69773 |
| 655 | 246 | 0.26 | 604 | 0 | 118 | 3.5 | 28 | 25 | 12 | 48.11535 |
| 655 | 246 | 0.26 | 604 | 0 | 118 | 3.5 | 28 | 25 | 12 | 36.58998 |
| 655 | 246 | 0.26 | 604 | 0 | 118 | 3.5 | 28 | 25 | 12 | 39.9639 |
| 655 | 246 | 0.26 | 604 | 0 | 118 | 3.5 | 28 | 25 | 12 | 35.63843 |
| 655 | 246 | 0.26 | 604 | 0 | 118 | 3.5 | 28 | 25 | 12 | 53.5186 |
| 655 | 246 | 0.26 | 604 | 0 | 118 | 3.5 | 28 | 25 | 12 | 50.47529 |
| 655 | 246 | 0.26 | 604 | 0 | 118 | 3.5 | 28 | 25 | 12 | 61.37422 |
| 655 | 246 | 0.26 | 604 | 0 | 118 | 3.5 | 28 | 25 | 12 | 60.87002 |
| 655 | 246 | 0.26 | 604 | 0 | 118 | 3.5 | 28 | 25 | 12 | 56.21109 |
| 655 | 246 | 0.26 | 604 | 0 | 118 | 3.5 | 28 | 25 | 12 | 35.77547 |
| 655 | 246 | 0.26 | 604 | 0 | 118 | 3.5 | 28 | 25 | 12 | 29.53142 |
| 655 | 246 | 0.26 | 604 | 0 | 118 | 3.5 | 28 | 25 | 12 | 42.24916 |
| 655 | 246 | 0.26 | 604 | 0 | 118 | 3.5 | 28 | 25 | 12 | 41.08497 |
| 655 | 246 | 0.26 | 604 | 0 | 118 | 3.5 | 28 | 25 | 12 | 36.49765 |
| 655 | 246 | 0.26 | 604 | 0 | 118 | 3.5 | 28 | 25 | 12 | 48.99541 |
| 655 | 246 | 0.26 | 604 | 0 | 118 | 3.5 | 28 | 25 | 12 | 43.3211 |
| 655 | 246 | 0.26 | 604 | 0 | 118 | 3.5 | 28 | 25 | 12 | 42.20553 |
| 655 | 246 | 0.26 | 604 | 0 | 118 | 3.5 | 28 | 25 | 12 | 47.62403 |
| 655 | 246 | 0.26 | 604 | 0 | 118 | 3.5 | 28 | 25 | 12 | 39.67172 |
| 655 | 246 | 0.26 | 604 | 0 | 118 | 3.5 | 28 | 25 | 12 | 45.1334 |
| 655 | 246 | 0.26 | 604 | 0 | 118 | 3.5 | 28 | 25 | 12 | 44.19032 |
| 655 | 246 | 0.26 | 604 | 0 | 118 | 3.5 | 28 | 25 | 12 | 36.46536 |
| 655 | 246 | 0.26 | 604 | 0 | 118 | 3.5 | 28 | 25 | 12 | 39.0354 |
| 655 | 246 | 0.26 | 604 | 0 | 118 | 3.5 | 28 | 25 | 12 | 44.76147 |
| 655 | 246 | 0.26 | 604 | 0 | 118 | 3.5 | 28 | 25 | 12 | 49.80695 |
| 655 | 246 | 0.26 | 604 | 0 | 118 | 3.5 | 28 | 25 | 12 | 44.33581 |
| 655 | 246 | 0.26 | 604 | 0 | 118 | 3.5 | 28 | 25 | 12 | 35.17062 |
| 655 | 246 | 0.26 | 604 | 0 | 118 | 3.5 | 28 | 25 | 12 | 30.48143 |
| 655 | 246 | 0.26 | 604 | 0 | 118 | 3.5 | 28 | 25 | 12 | 30.59874 |
| 655 | 246 | 0.26 | 604 | 0 | 118 | 3.5 | 28 | 25 | 12 | 49.85146 |
| 655 | 246 | 0.26 | 604 | 0 | 118 | 3.5 | 28 | 25 | 12 | 43.33562 |
| 655 | 246 | 0.26 | 604 | 0 | 118 | 3.5 | 28 | 25 | 12 | 44.2 |
| 655 | 246 | 0.26 | 604 | 0 | 118 | 3.5 | 28 | 25 | 12 | 34 |
| 655 | 246 | 0.26 | 604 | 0 | 118 | 3.5 | 28 | 25 | 12 | 53.6423 |
| 655 | 246 | 0.26 | 604 | 0 | 118 | 3.5 | 28 | 25 | 12 | 38.20382 |
| 655 | 246 | 0.26 | 604 | 0 | 118 | 3.5 | 28 | 25 | 12 | 48.44283 |
| 655 | 246 | 0.26 | 604 | 0 | 118 | 3.5 | 28 | 25 | 12 | 47.84551 |
| 655 | 246 | 0.26 | 604 | 0 | 118 | 3.5 | 28 | 25 | 12 | 38.77388 |
| 655 | 246 | 0.26 | 604 | 0 | 118 | 3.5 | 28 | 25 | 12 | 34.5 |
| 655 | 246 | 0.26 | 604 | 0 | 118 | 3.5 | 28 | 25 | 12 | 35.3 |
| 483 | 1074 | 0.17 | 0 | 322 | 268 | 10.7 | 28 | 20 | 3 | 88 |
| 483 | 1074 | 0.17 | 0 | 322 | 268 | 10.7 | 28 | 20 | 3 | 90 |
| 483 | 1074 | 0.17 | 0 | 322 | 268 | 10.7 | 28 | 20 | 3 | 95 |
| 483 | 1074 | 0.17 | 0 | 322 | 268 | 10.7 | 28 | 20 | 3 | 92 |
| 483 | 1074 | 0.17 | 0 | 322 | 268 | 10.7 | 28 | 20 | 3 | 93 |
| 483 | 1074 | 0.17 | 0 | 322 | 268 | 10.7 | 28 | 20 | 3 | 97 |
| 483 | 1074 | 0.17 | 0 | 322 | 268 | 10.7 | 28 | 20 | 3 | 87 |
| 483 | 1074 | 0.17 | 0 | 322 | 268 | 10.7 | 28 | 20 | 3 | 91 |
| 483 | 1074 | 0.17 | 0 | 322 | 268 | 10.7 | 28 | 20 | 3 | 95 |
| 483 | 1074 | 0.17 | 0 | 322 | 268 | 10.7 | 28 | 20 | 3 | 102 |
| 483 | 1074 | 0.17 | 0 | 322 | 268 | 10.7 | 28 | 20 | 3 | 113 |
| 483 | 1074 | 0.17 | 0 | 322 | 268 | 10.7 | 28 | 20 | 3 | 70 |
| 483 | 1074 | 0.17 | 0 | 322 | 268 | 10.7 | 28 | 20 | 3 | 116 |
| 483 | 1074 | 0.17 | 0 | 322 | 268 | 10.7 | 28 | 20 | 3 | 114 |
| 480 | 1074 | 0.17 | 0 | 322 | 268 | 10.7 | 28 | 20 | 3 | 75 |
| 480 | 1074 | 0.17 | 0 | 322 | 268 | 10.7 | 28 | 20 | 3 | 98 |
| 480 | 1074 | 0.17 | 0 | 322 | 268 | 10.7 | 28 | 20 | 3 | 110 |
| 480 | 1074 | 0.17 | 0 | 322 | 268 | 10.7 | 28 | 20 | 3 | 68 |
| 480 | 1074 | 0.17 | 0 | 322 | 268 | 10.7 | 28 | 20 | 3 | 116 |
| 480 | 1074 | 0.17 | 0 | 322 | 268 | 10.7 | 28 | 20 | 3 | 114 |
| 480 | 1074 | 0.17 | 0 | 322 | 268 | 10.7 | 28 | 20 | 3 | 75 |
| 480 | 1074 | 0.17 | 0 | 322 | 268 | 10.7 | 28 | 20 | 3 | 98 |
| 480 | 1074 | 0.17 | 0 | 322 | 268 | 10.7 | 28 | 20 | 3 | 108 |
| 480 | 1074 | 0.17 | 0 | 322 | 268 | 10.7 | 28 | 20 | 3 | 92 |
| 480 | 1074 | 0.17 | 0 | 322 | 268 | 10.7 | 28 | 20 | 3 | 83 |
| 480 | 1074 | 0.17 | 0 | 322 | 268 | 10.7 | 28 | 20 | 3 | 103 |
| 480 | 1074 | 0.17 | 0 | 322 | 268 | 10.7 | 28 | 20 | 3 | 78 |
| 483 | 1074 | 0.17 | 0 | 322 | 268 | 10.7 | 28 | 20 | 3 | 95 |
| 483 | 1074 | 0.17 | 0 | 322 | 268 | 10.7 | 28 | 20 | 3 | 98 |
| 483 | 1074 | 0.17 | 0 | 322 | 268 | 10.7 | 28 | 20 | 3 | 82 |
| 483 | 1074 | 0.17 | 0 | 322 | 268 | 10.7 | 28 | 20 | 3 | 99 |
| 483 | 1074 | 0.17 | 0 | 322 | 268 | 10.7 | 28 | 20 | 3 | 99 |
| 483 | 1074 | 0.17 | 0 | 322 | 268 | 10.7 | 28 | 20 | 3 | 85 |
| 483 | 1074 | 0.17 | 0 | 322 | 268 | 10.7 | 28 | 20 | 3 | 95 |
| 483 | 1074 | 0.17 | 0 | 322 | 268 | 10.7 | 28 | 20 | 3 | 100 |
| 483 | 1074 | 0.17 | 0 | 322 | 268 | 10.7 | 28 | 20 | 3 | 83 |
| 483 | 1074 | 0.17 | 0 | 322 | 268 | 10.7 | 28 | 20 | 3 | 85 |
| 483 | 1074 | 0.17 | 0 | 322 | 268 | 10.7 | 28 | 20 | 3 | 102 |
| 483 | 1074 | 0.17 | 0 | 322 | 268 | 10.7 | 28 | 20 | 3 | 78 |
| 483 | 1074 | 0.17 | 0 | 322 | 268 | 10.7 | 28 | 20 | 3 | 87 |
| 483 | 1074 | 0.17 | 0 | 322 | 268 | 10.7 | 28 | 20 | 3 | 104 |
| 483 | 1074 | 0.17 | 0 | 322 | 268 | 10.7 | 28 | 20 | 3 | 85 |
| 483 | 1074 | 0.17 | 0 | 322 | 268 | 10.7 | 28 | 20 | 3 | 83 |
| 483 | 1074 | 0.17 | 0 | 322 | 268 | 10.7 | 28 | 20 | 3 | 100 |
| 483 | 1074 | 0.17 | 0 | 322 | 268 | 10.7 | 28 | 20 | 3 | 76 |
| 483 | 1074 | 0.17 | 0 | 322 | 268 | 10.7 | 28 | 20 | 6 | 88 |
| 483 | 1074 | 0.17 | 0 | 322 | 268 | 10.7 | 28 | 20 | 6 | 90 |
| 483 | 1074 | 0.17 | 0 | 322 | 268 | 10.7 | 28 | 20 | 6 | 95 |
| 483 | 1074 | 0.17 | 0 | 322 | 268 | 10.7 | 28 | 20 | 6 | 91 |
| 483 | 1074 | 0.17 | 0 | 322 | 268 | 10.7 | 28 | 20 | 6 | 91 |
| 483 | 1074 | 0.17 | 0 | 322 | 268 | 10.7 | 28 | 20 | 6 | 96 |
| 483 | 1074 | 0.17 | 0 | 322 | 268 | 10.7 | 28 | 20 | 6 | 85 |
| 483 | 1074 | 0.17 | 0 | 322 | 268 | 10.7 | 28 | 20 | 6 | 89 |
| 483 | 1074 | 0.17 | 0 | 322 | 268 | 10.7 | 28 | 20 | 6 | 94 |
| 483 | 1074 | 0.17 | 0 | 322 | 268 | 10.7 | 28 | 20 | 6 | 105 |
| 483 | 1074 | 0.17 | 0 | 322 | 268 | 10.7 | 28 | 20 | 6 | 85 |
| 483 | 1074 | 0.17 | 0 | 322 | 268 | 10.7 | 28 | 20 | 6 | 96 |
| 483 | 1074 | 0.17 | 0 | 322 | 268 | 10.7 | 28 | 20 | 6 | 106 |
| 483 | 1074 | 0.17 | 0 | 322 | 268 | 10.7 | 28 | 20 | 6 | 86.5 |
| 483 | 1074 | 0.17 | 0 | 322 | 268 | 10.7 | 28 | 20 | 6 | 98 |
| 483 | 1074 | 0.17 | 0 | 322 | 268 | 10.7 | 28 | 20 | 6 | 104.3 |
| 483 | 1074 | 0.17 | 0 | 322 | 268 | 10.7 | 28 | 20 | 6 | 83.8 |
| 483 | 1074 | 0.17 | 0 | 322 | 268 | 10.7 | 28 | 20 | 6 | 94 |
| 483 | 1074 | 0.17 | 0 | 322 | 268 | 10.7 | 28 | 20 | 6 | 88 |
| 483 | 1074 | 0.17 | 0 | 322 | 268 | 10.7 | 28 | 20 | 6 | 92 |
| 483 | 1074 | 0.17 | 0 | 322 | 268 | 10.7 | 28 | 20 | 6 | 107 |
| 483 | 1074 | 0.17 | 0 | 322 | 268 | 10.7 | 28 | 20 | 6 | 88.5 |
| 483 | 1074 | 0.17 | 0 | 322 | 268 | 10.7 | 28 | 20 | 6 | 93 |
| 483 | 1074 | 0.17 | 0 | 322 | 268 | 10.7 | 28 | 20 | 6 | 108.5 |
| 483 | 1074 | 0.17 | 0 | 322 | 268 | 10.7 | 28 | 20 | 6 | 87.5 |
| 483 | 1074 | 0.17 | 0 | 322 | 268 | 10.7 | 28 | 20 | 6 | 91.2 |
| 483 | 1074 | 0.17 | 0 | 322 | 268 | 10.7 | 28 | 20 | 6 | 105.5 |
| 483 | 1074 | 0.17 | 0 | 322 | 268 | 10.7 | 28 | 20 | 6 | 108 |
| 483 | 1074 | 0.17 | 0 | 322 | 268 | 10.7 | 28 | 20 | 6 | 95 |
| 483 | 1074 | 0.17 | 0 | 322 | 268 | 10.7 | 28 | 20 | 6 | 96 |
| 483 | 1074 | 0.17 | 0 | 322 | 268 | 10.7 | 28 | 20 | 6 | 85 |
| 483 | 1074 | 0.17 | 0 | 322 | 268 | 10.7 | 28 | 20 | 6 | 89 |
| 483 | 1074 | 0.17 | 0 | 322 | 268 | 10.7 | 28 | 20 | 6 | 102 |
| 483 | 1074 | 0.17 | 0 | 322 | 268 | 10.7 | 28 | 20 | 6 | 109.2 |
| 483 | 1074 | 0.17 | 0 | 322 | 268 | 10.7 | 28 | 20 | 6 | 96.3 |
| 309 | 345 | 0.24 | 1026 | 0 | 0 | 3 | 1 | 39 | 8 | 8 |
| 309 | 345 | 0.24 | 1026 | 0 | 0 | 3 | 3 | 39 | 8 | 15 |
| 309 | 345 | 0.24 | 1026 | 0 | 0 | 3 | 3 | 39 | 8 | 16 |
| 309 | 345 | 0.24 | 1026 | 0 | 0 | 3 | 3 | 39 | 8 | 13.7 |
| 309 | 345 | 0.24 | 1026 | 0 | 0 | 3 | 7 | 39 | 8 | 21 |
| 309 | 345 | 0.24 | 1026 | 0 | 0 | 3 | 7 | 39 | 8 | 25 |
| 309 | 345 | 0.24 | 1026 | 0 | 0 | 3 | 7 | 39 | 8 | 19 |
| 309 | 345 | 0.24 | 1026 | 0 | 0 | 3 | 28 | 39 | 8 | 24 |
| 309 | 345 | 0.24 | 1026 | 0 | 0 | 3 | 28 | 39 | 8 | 27 |
| 309 | 345 | 0.24 | 1026 | 0 | 0 | 3 | 28 | 39 | 8 | 21 |
| 309 | 345 | 0.24 | 1026 | 0 | 0 | 3 | 1 | 39 | 8 | 11 |
| 309 | 345 | 0.24 | 1026 | 0 | 0 | 3 | 1 | 39 | 8 | 10 |
| 309 | 345 | 0.24 | 1026 | 0 | 0 | 3 | 1 | 39 | 8 | 9.5 |
| 309 | 345 | 0.24 | 1026 | 0 | 0 | 3 | 3 | 39 | 8 | 18 |
| 309 | 345 | 0.24 | 1026 | 0 | 0 | 3 | 3 | 39 | 8 | 17 |
| 309 | 345 | 0.24 | 1026 | 0 | 0 | 3 | 3 | 39 | 8 | 15 |
| 309 | 345 | 0.24 | 1026 | 0 | 0 | 3 | 3 | 39 | 8 | 16 |
| 309 | 345 | 0.24 | 1026 | 0 | 0 | 3 | 3 | 39 | 8 | 13.7 |
| 309 | 345 | 0.24 | 1026 | 0 | 0 | 3 | 7 | 39 | 8 | 21 |
| 309 | 345 | 0.24 | 1026 | 0 | 0 | 3 | 7 | 39 | 8 | 25 |
| 309 | 345 | 0.24 | 1026 | 0 | 0 | 3 | 7 | 39 | 8 | 19 |
| 309 | 345 | 0.24 | 1026 | 0 | 0 | 3 | 28 | 39 | 8 | 24 |
| 309 | 345 | 0.24 | 1026 | 0 | 0 | 3 | 28 | 39 | 8 | 27 |
| 309 | 345 | 0.24 | 1026 | 0 | 0 | 3 | 28 | 39 | 8 | 21 |
| 309 | 345 | 0.24 | 1026 | 0 | 0 | 3 | 1 | 39 | 8 | 11 |
| 309 | 345 | 0.24 | 1026 | 0 | 0 | 3 | 1 | 39 | 8 | 10 |
| 309 | 345 | 0.24 | 1026 | 0 | 0 | 3 | 1 | 39 | 8 | 9.5 |
| 309 | 345 | 0.24 | 1026 | 0 | 0 | 3 | 3 | 39 | 8 | 18 |
| 309 | 345 | 0.24 | 1026 | 0 | 0 | 3 | 3 | 39 | 8 | 17 |
| 309 | 345 | 0.24 | 1026 | 0 | 0 | 3 | 3 | 39 | 8 | 16.4 |
| 309 | 345 | 0.24 | 1026 | 0 | 0 | 3 | 7 | 39 | 8 | 24 |
| 309 | 345 | 0.24 | 1026 | 0 | 0 | 3 | 7 | 39 | 8 | 25 |
| 309 | 345 | 0.24 | 1026 | 0 | 0 | 3 | 7 | 39 | 8 | 25.9 |
| 309 | 345 | 0.24 | 1026 | 0 | 0 | 3 | 28 | 39 | 8 | 29 |
| 309 | 345 | 0.24 | 1026 | 0 | 0 | 3 | 7 | 39 | 8 | 21 |
| 309 | 345 | 0.24 | 1026 | 0 | 0 | 3 | 7 | 39 | 8 | 25 |
| 309 | 345 | 0.24 | 1026 | 0 | 0 | 3 | 7 | 39 | 8 | 19 |
| 309 | 345 | 0.24 | 1026 | 0 | 0 | 3 | 28 | 39 | 8 | 24 |
| 309 | 345 | 0.24 | 1026 | 0 | 0 | 3 | 28 | 39 | 8 | 27 |
| 309 | 345 | 0.24 | 1026 | 0 | 0 | 3 | 28 | 39 | 8 | 21 |
| 309 | 345 | 0.24 | 1026 | 0 | 0 | 3 | 1 | 39 | 8 | 11 |
| 309 | 345 | 0.24 | 1026 | 0 | 0 | 3 | 1 | 39 | 8 | 10 |
| 309 | 345 | 0.24 | 1026 | 0 | 0 | 3 | 1 | 39 | 8 | 9.5 |
| 309 | 345 | 0.24 | 1026 | 0 | 0 | 3 | 3 | 39 | 8 | 18 |
| 309 | 345 | 0.24 | 1026 | 0 | 0 | 3 | 3 | 39 | 8 | 17 |
| 309 | 345 | 0.24 | 1026 | 0 | 0 | 3 | 3 | 39 | 8 | 16.4 |
| 309 | 345 | 0.24 | 1026 | 0 | 0 | 3 | 7 | 39 | 8 | 24 |
| 309 | 345 | 0.24 | 1026 | 0 | 0 | 3 | 28 | 39 | 8 | 27.2 |
| 309 | 345 | 0.24 | 1026 | 0 | 0 | 3 | 28 | 39 | 8 | 26.3 |
| 309 | 345 | 0.24 | 1026 | 0 | 0 | 3 | 1 | 39 | 8 | 9 |
| 309 | 345 | 0.24 | 1026 | 0 | 0 | 3 | 1 | 39 | 8 | 8.5 |
| 309 | 345 | 0.24 | 1026 | 0 | 0 | 3 | 1 | 39 | 8 | 9.4 |
| 309 | 345 | 0.24 | 1026 | 0 | 0 | 3 | 3 | 39 | 8 | 15 |
| 309 | 345 | 0.24 | 1026 | 0 | 0 | 3 | 3 | 39 | 8 | 15.8 |
| 309 | 345 | 0.24 | 1026 | 0 | 0 | 3 | 3 | 39 | 8 | 14 |
| 309 | 345 | 0.24 | 1026 | 0 | 0 | 3 | 7 | 39 | 8 | 18.2 |
| 309 | 345 | 0.24 | 1026 | 0 | 0 | 3 | 7 | 39 | 8 | 21 |
| 309 | 345 | 0.24 | 1026 | 0 | 0 | 3 | 7 | 39 | 8 | 16.4 |
| 309 | 345 | 0.24 | 1026 | 0 | 0 | 3 | 28 | 39 | 8 | 21.2 |
| 309 | 345 | 0.24 | 1026 | 0 | 0 | 3 | 28 | 39 | 8 | 20.4 |
| 309 | 345 | 0.24 | 1026 | 0 | 0 | 3 | 28 | 39 | 8 | 19.5 |
| 483 | 1074 | 0.17 | 0 | 322 | 268 | 0 | 28 | 39 | 6 | 88 |
| 483 | 1074 | 0.17 | 0 | 322 | 268 | 0 | 28 | 39 | 6 | 92 |
| 483 | 1074 | 0.17 | 0 | 322 | 268 | 0 | 28 | 39 | 6 | 86 |
| 483 | 1074 | 0.17 | 0 | 322 | 268 | 0 | 28 | 39 | 6 | 94.3 |
| 483 | 1074 | 0.17 | 0 | 322 | 268 | 0 | 28 | 39 | 6 | 91.2 |
| 483 | 1074 | 0.17 | 0 | 322 | 268 | 0 | 28 | 39 | 6 | 90.1 |
| 483 | 1074 | 0.17 | 0 | 322 | 268 | 0 | 28 | 39 | 6 | 89.3 |
| 483 | 1074 | 0.17 | 0 | 322 | 268 | 0 | 28 | 39 | 6 | 74.6 |
| 483 | 1074 | 0.17 | 0 | 322 | 268 | 0 | 28 | 39 | 6 | 75.5 |
| 483 | 1074 | 0.17 | 0 | 322 | 268 | 0 | 28 | 39 | 6 | 80.1 |
| 483 | 1074 | 0.17 | 0 | 322 | 268 | 0 | 28 | 39 | 6 | 96.2 |
| 483 | 1074 | 0.17 | 0 | 322 | 268 | 0 | 28 | 39 | 6 | 75.1 |
| 483 | 1074 | 0.17 | 0 | 322 | 268 | 0 | 28 | 39 | 6 | 76.4 |
| 483 | 1074 | 0.17 | 0 | 322 | 268 | 0 | 28 | 39 | 6 | 80.1 |
| 483 | 1074 | 0.17 | 0 | 322 | 268 | 0 | 28 | 39 | 6 | 79.2 |
| 483 | 1074 | 0.17 | 0 | 322 | 268 | 0 | 28 | 39 | 6 | 88 |
| 483 | 1074 | 0.17 | 0 | 322 | 268 | 0 | 28 | 39 | 6 | 80 |
| 483 | 1074 | 0.17 | 0 | 322 | 268 | 0 | 28 | 39 | 6 | 78.2 |
| 483 | 1074 | 0.17 | 0 | 322 | 268 | 0 | 28 | 39 | 6 | 90.3 |
| 483 | 1074 | 0.17 | 0 | 322 | 268 | 0 | 28 | 39 | 6 | 88.3 |
| 483 | 1074 | 0.17 | 0 | 322 | 268 | 0 | 28 | 39 | 6 | 90.4 |
| 483 | 1074 | 0.17 | 0 | 322 | 268 | 0 | 28 | 39 | 6 | 63.1 |
| 483 | 1074 | 0.17 | 0 | 322 | 268 | 0 | 28 | 39 | 6 | 73.2 |
| 483 | 1074 | 0.17 | 0 | 322 | 268 | 0 | 28 | 39 | 6 | 84.6 |
| 483 | 1074 | 0.17 | 0 | 322 | 268 | 0 | 28 | 39 | 6 | 92.3 |
| 483 | 1074 | 0.17 | 0 | 322 | 268 | 0 | 28 | 39 | 6 | 97.4 |
| 483 | 1074 | 0.17 | 0 | 322 | 268 | 0 | 28 | 39 | 6 | 75.6 |
| 483 | 1074 | 0.17 | 0 | 322 | 268 | 0 | 28 | 39 | 6 | 69.2 |
| 483 | 1074 | 0.17 | 0 | 322 | 268 | 0 | 28 | 39 | 6 | 69 |
| 483 | 1074 | 0.17 | 0 | 322 | 268 | 0 | 28 | 39 | 6 | 89.5 |
| 1112.3 | 1902 | 0.27 | 322.6 | 0 | 155.7 | 20 | 28 | 15 | 18 | 34.23 |
| 1112.3 | 1902 | 0.27 | 322.6 | 0 | 155.7 | 20 | 28 | 15 | 18 | 36.45 |
| 1112.3 | 1902 | 0.27 | 322.6 | 0 | 155.7 | 20 | 28 | 15 | 18 | 26.54 |
| 1112.3 | 1902 | 0.27 | 322.6 | 0 | 155.7 | 20 | 28 | 15 | 18 | 40.12 |
| 1112.3 | 1902 | 0.27 | 322.6 | 0 | 155.7 | 20 | 28 | 15 | 18 | 37.54 |
| 1112.3 | 1902 | 0.27 | 322.6 | 0 | 155.7 | 20 | 28 | 15 | 18 | 32.14 |
| 1112.3 | 1902 | 0.27 | 322.6 | 0 | 155.7 | 20 | 28 | 15 | 18 | 45.98 |
| 1112.3 | 1902 | 0.27 | 322.6 | 0 | 155.7 | 20 | 28 | 15 | 18 | 35.84 |
| 1112.3 | 1902 | 0.27 | 322.6 | 0 | 155.7 | 20 | 28 | 15 | 18 | 34.62 |
| 285.3 | 285.3 | 0.30 | 1141.1 | 0 | 0 | 5.3 | 7 | 39 | 12 | 16.45 |
| 285.3 | 285.3 | 0.30 | 1141.1 | 0 | 0 | 5.3 | 7 | 39 | 12 | 16.76 |
| 285.3 | 285.3 | 0.30 | 1141.1 | 0 | 0 | 5.3 | 7 | 39 | 12 | 16.2 |
| 285.3 | 285.3 | 0.30 | 1141.1 | 0 | 0 | 5.3 | 7 | 39 | 12 | 18.68 |
| 285.3 | 285.3 | 0.30 | 1141.1 | 0 | 0 | 5.3 | 7 | 39 | 12 | 19.03 |
| 285.3 | 285.3 | 0.30 | 1141.1 | 0 | 0 | 5.3 | 7 | 39 | 12 | 18.18 |
| 881.6 | 881.6 | 0.16 | 0 | 0 | 377.8 | 12.6 | 28 | 200 | 6 | 131.2 |
| 881.6 | 881.6 | 0.16 | 0 | 0 | 377.8 | 12.6 | 28 | 200 | 6 | 120.5 |
| 881.6 | 881.6 | 0.16 | 0 | 0 | 377.8 | 12.6 | 28 | 200 | 6 | 126.3 |
| 881.6 | 881.6 | 0.16 | 0 | 0 | 377.8 | 12.6 | 28 | 200 | 6 | 150.6 |
| 881.6 | 881.6 | 0.16 | 0 | 0 | 377.8 | 12.6 | 28 | 200 | 6 | 140.3 |
| 881.6 | 881.6 | 0.16 | 0 | 0 | 377.8 | 12.6 | 28 | 200 | 6 | 145.6 |
| 881.6 | 881.6 | 0.16 | 0 | 0 | 377.8 | 12.6 | 28 | 200 | 6 | 153.4 |
| 881.6 | 881.6 | 0.16 | 0 | 0 | 377.8 | 12.6 | 28 | 200 | 6 | 151.1 |
| 881.6 | 881.6 | 0.16 | 0 | 0 | 377.8 | 12.6 | 28 | 200 | 6 | 152.5 |
| 881.6 | 881.6 | 0.16 | 0 | 0 | 377.8 | 12.6 | 28 | 200 | 6 | 120.3 |
| 881.6 | 881.6 | 0.16 | 0 | 0 | 377.8 | 12.6 | 28 | 200 | 6 | 114.6 |
| 881.6 | 881.6 | 0.16 | 0 | 0 | 377.8 | 12.6 | 28 | 200 | 6 | 113.5 |
| 881.6 | 881.6 | 0.16 | 0 | 0 | 377.8 | 12.6 | 28 | 200 | 6 | 136.2 |
| 881.6 | 881.6 | 0.16 | 0 | 0 | 377.8 | 12.6 | 28 | 200 | 6 | 129.6 |
| 881.6 | 881.6 | 0.16 | 0 | 0 | 377.8 | 12.6 | 28 | 200 | 6 | 132.1 |
| 881.6 | 881.6 | 0.16 | 0 | 0 | 377.8 | 12.6 | 28 | 200 | 6 | 136.9 |
| 881.6 | 881.6 | 0.16 | 0 | 0 | 377.8 | 12.6 | 28 | 200 | 6 | 126.8 |
| 881.6 | 881.6 | 0.16 | 0 | 0 | 377.8 | 12.6 | 28 | 200 | 6 | 133.7 |
| 881.6 | 881.6 | 0.16 | 0 | 0 | 377.8 | 12.6 | 28 | 200 | 6 | 118.6 |
| 881.6 | 881.6 | 0.16 | 0 | 0 | 377.8 | 12.6 | 28 | 200 | 6 | 105.4 |
| 881.6 | 881.6 | 0.16 | 0 | 0 | 377.8 | 12.6 | 28 | 200 | 6 | 110.1 |
| 881.6 | 881.6 | 0.16 | 0 | 0 | 377.8 | 12.6 | 28 | 200 | 6 | 129.2 |
| 881.6 | 881.6 | 0.16 | 0 | 0 | 377.8 | 12.6 | 28 | 200 | 6 | 123.5 |
| 881.6 | 881.6 | 0.16 | 0 | 0 | 377.8 | 12.6 | 28 | 200 | 6 | 126.1 |
| 881.6 | 881.6 | 0.16 | 0 | 0 | 377.8 | 12.6 | 28 | 200 | 6 | 141.9 |
| 881.6 | 881.6 | 0.16 | 0 | 0 | 377.8 | 12.6 | 28 | 200 | 6 | 138.5 |
| 881.6 | 881.6 | 0.16 | 0 | 0 | 377.8 | 12.6 | 28 | 200 | 6 | 140.7 |
| 450 | 1085 | 0.35 | 0 | 450 | 0 | 3.78 | 28 | 35 | 0 | 35.2 |
| 450 | 1085 | 0.35 | 0 | 450 | 0 | 3.78 | 28 | 35 | 0 | 35.2 |
| 450 | 1085 | 0.35 | 0 | 450 | 0 | 3.78 | 28 | 35 | 0 | 41.2 |
| 450 | 1085 | 0.35 | 0 | 450 | 0 | 3.78 | 28 | 35 | 0 | 28.1 |
| 450 | 1085 | 0.35 | 0 | 450 | 0 | 3.78 | 28 | 35 | 3 | 40.2 |
| 450 | 1085 | 0.35 | 0 | 450 | 0 | 3.78 | 28 | 35 | 3 | 36.1 |
| 450 | 1085 | 0.35 | 0 | 450 | 0 | 3.78 | 28 | 35 | 3 | 30 |
| 450 | 1085 | 0.35 | 0 | 450 | 0 | 3.78 | 28 | 35 | 3 | 38.5 |
| 450 | 1085 | 0.35 | 0 | 450 | 0 | 3.78 | 28 | 35 | 3 | 28.6 |
| 450 | 1085 | 0.35 | 0 | 450 | 0 | 3.78 | 28 | 35 | 3 | 34.3 |
| 450 | 1085 | 0.35 | 0 | 450 | 0 | 3.78 | 28 | 35 | 6 | 38.2 |
| 450 | 1085 | 0.35 | 0 | 450 | 0 | 3.78 | 28 | 35 | 6 | 41.6 |
| 450 | 1085 | 0.35 | 0 | 450 | 0 | 3.78 | 28 | 35 | 6 | 35.6 |
| 450 | 1085 | 0.35 | 0 | 450 | 0 | 3.78 | 28 | 35 | 6 | 38.2 |
| 450 | 1085 | 0.35 | 0 | 450 | 0 | 3.78 | 28 | 35 | 6 | 27.3 |
| 450 | 1085 | 0.35 | 0 | 450 | 0 | 3.78 | 28 | 35 | 6 | 33.2 |
| 483 | 1074 | 0.17 | 0 | 322 | 268 | 0 | 28 | 39 | 6 | 84.6 |
| 483 | 1074 | 0.17 | 0 | 322 | 268 | 0 | 28 | 39 | 6 | 92.3 |
| 483 | 1074 | 0.17 | 0 | 322 | 268 | 0 | 28 | 39 | 6 | 97.4 |
| 483 | 1074 | 0.17 | 0 | 322 | 268 | 0 | 28 | 39 | 6 | 75.6 |
| 483 | 1074 | 0.17 | 0 | 322 | 268 | 0 | 28 | 39 | 6 | 69.2 |
| 483 | 1074 | 0.17 | 0 | 322 | 268 | 0 | 28 | 39 | 6 | 69 |
| 483 | 1074 | 0.17 | 0 | 322 | 268 | 0 | 28 | 39 | 6 | 89.5 |
